# New insight into the biological activity of *Salmo salar* NK-lysin antimicrobial peptides

**DOI:** 10.3389/fimmu.2024.1191966

**Published:** 2024-04-09

**Authors:** Leonardo Ortega, Crisleri Carrera, Carolina Muñoz-Flores, Santiago Salazar, Milton F. Villegas, María F. Starck, Ariel Valenzuela, Niza Agurto, Raquel Montesino, Allisson Astuya, Natalie Parra, Ercilia T. Pérez, Natacha Santibáñez, Alex Romero, Pamela Ruíz, Emilio Lamazares, Fátima Reyes, Oliberto Sánchez, Jorge R. Toledo, Jannel Acosta

**Affiliations:** ^1^ Laboratorio de Biotecnología y Biofármacos, Departamento de Fisiopatología, Facultad de Ciencias Biológicas, Universidad de Concepción, Concepción, Chile; ^2^ Laboratorio de Piscicultura y Patología Acuática, Departamento de Oceanografía, Facultad de Ciencias Naturales y Oceanográficas, Universidad de Concepción, Concepción, Chile; ^3^ Laboratorio de Genómica Marina y Cultivo Celular, Departamento de Oceanografía y Centro de Investigación Oceanográfica en el Pacífico Sur Oriental (COPAS) Sur-Austral, Facultad de Ciencias Naturales y Oceanográficas, Universidad de Concepción, Concepción, Chile; ^4^ Laboratorio de Inmunología y Estrés de Organismos Acuáticos, Instituto de Patología Animal, Facultad de Ciencias Veterinarias, Universidad Austral de Chile, Valdivia, Chile; ^5^ Centro Fondo de Financiamiento de Centros de Investigación en Áreas Prioritarias (FONDAP), Interdisciplinary Center for Aquaculture Research (INCAR), Universidad de Concepción, Concepción, Chile; ^6^ Departamento de Ciencias Biológicas, Facultad de Ciencias de la Vida, Universidad Andrés Bello, Talcahuano, Chile; ^7^ Laboratorio de Biofármacos Recombinantes, Departamento de Farmacología, Facultad de Ciencias Biológicas, Universidad de Concepción, Concepción, Chile

**Keywords:** NK-lysin, antimicrobial peptide, immune response, cytokines, Salmo salar

## Abstract

NK-lysin is a potent antimicrobial peptide (AMP) with antimicrobial activity against bacteria, fungi, viruses, and parasites. NK-lysin is a type of granulysin, a member of the saposin-like proteins family first isolated from a pig’s small intestine. In previous work, for the first time, we identified four variants of *nk-lysin* from Atlantic salmon (*Salmo salar*) using EST sequences. In the present study, we reported and characterized two additional transcripts of *NK-lysin* from *S. salar*. Besides, we evaluated the tissue distribution of three *NK-lysins* from *S. salar* and assessed the antimicrobial, hemolytic, and immunomodulatory activities and signaling pathways of three NK-lysin-derived peptides. The synthetic peptides displayed antimicrobial activity against *Piscirickettsia salmonis* (LF-89) and *Flavobacterium psychrophilum*. These peptides induced the expression of immune genes related to innate and adaptive immune responses *in vitro* and *in vivo*. The immunomodulatory activity of the peptides involves the mitogen-activated protein kinases-mediated signaling pathway, including p38, extracellular signal-regulated kinase 1/2, and/or c-Jun N-terminal kinases. Besides, the peptides modulated the immune response induced by pathogen-associated molecular patterns (PAMPs). Our findings show that NK-lysin could be a highly effective immunostimulant or vaccine adjuvant for use in fish aquaculture.

## 1 Introduction

Aquaculture is an important economic sector, but it is constantly threatened by infectious diseases (1). Intensification of aquaculture production increases fish’s susceptibility to infectious diseases due to immunosuppression, increasing mortality. Therefore, knowledge of the organization and function of the fish immune system is essential to promote aquaculture as an economic activity.

Antimicrobial peptides (AMP) are small, amphipathic molecules that play a crucial role in innate immunity (2–4) and have been isolated from insects (5), amphibians (6, 7), mammals (8), bacteria (9), and fish (4). Most of these peptides show antibacterial, antiviral, antifungal, and antitumor activity, in addition to immunomodulatory functions. AMPs can connect innate and adaptive immunity, impacting immune response quality, efficacy, and direction (9). Regarding their immunomodulatory activity, these peptides induce chemokine and cytokine production, pro/anti-inflammatory activity, direct chemotaxis, wound healing, angiogenesis, apoptotic activity, and adjuvant activity (10–12). AMPs include NK-lysin, which is a type of granulysin first isolated from a pig small intestine and identified as a peptide effector of cytotoxic T lymphocytes (CTL) and natural killer (NK) cells with antimicrobial properties (13). It is present on CD8^+^, CD2^+,^ and CD4^+^ cells and is produced by CTLs and NK cells after overstimulation with IL-2. These peptides have 74-78 amino acid residues and six conserved cysteine residues, forming three disulfide bonds (13).

In teleosts, NK-lysin has been identified in *Ictalurus punctatus* (14), *Paralichthys olivaceus* (15), *Cynoglossus semilaevis* (16), *Takifugu rubripes* (GenBank Accession Number XP_003962755), *Salmo salar* (17), *Larimichthys crocea* (18), *Danio rerio* (19), *Cyprinus carpio* (20), *Oreochromis niloticus* (21), *Oncorhynchus mykiss* (22), *Trematomus bernacchii* (23), *Boleophthalmus pectinirostris* (24), *Thamnaconus modestus* (25), *Hyporthodus septemfasciatus* (26), *Dicentrarchus labrax* (27), *Trachinotus ovatus* (28), *Nibea albiflora* (29), *Sebastes schlegelii* (30), *Takifugu obscurus* (31), and *Scophthalmus maximus* (GenBank Accession Number KU705506.1), among others. Many studies focus on NK-lysins gene expression in different tissues under normal conditions and after exposure to a specific pathogen. These expression studies suggest NK-lysin involvement in the host immune response during bacterial and viral infection (14, 16). In addition, NK-lysins have direct antimicrobial activity against viral and bacterial pathogens and immunomodulatory functions, adding a new dimension to the classical role of NK-lysin as an antimicrobial, mainly against bacteria and parasites (16).

As mammalian, fish NK-lysins possess the conserved SapB domain that adopts an α-helix structure. Several peptides derived from the SapB domain of NK-lysin have been synthesized and characterized. These synthetic peptides have shown antimicrobial activity against several pathogens (15, 32, 33). Besides, it was demonstrated that some of these peptides interact with target bacterial cells, destroy cell membrane integrity, penetrate the cytoplasm, and induce genomic DNA degradation (33).

The biological functions of NK-lysin, such as antibacterial (18, 20, 21, 24, 34–36), antiviral (16, 27, 30, 33, 37–39), and antiparasitic (40) activities, have been established in teleosts. However, in salmonids, information on the immunomodulatory effect of NK-lysin is scarce (41). Recently, a study characterized the expression of 6 NK-lysin variants in rainbow trout and observed modest up-regulation (2-3-fold) of five (*nkl 2-4* and rainbow trout *nkl-like a & b*) of the six NK-lysins in trout fry exposed to *Flavobacterium psychrophilum* infection at 5 days post-challenge (22). In addition, we previously identified and characterized for the first time four NK-lysin-like transcripts from Atlantic salmon (*S. salar*) based on EST sequences (17). By alignment between the NK-lysin sequences identified in *S. salar* and NKLP27, a peptide derived from *C. semilaevis* NK-lysin (33), we designed and synthesized two 27-amino acid peptides and evaluated whether these peptides modulate the immune response in *S. salar* head kidney leukocytes. These peptides induced the immune response in *S. salar* head kidney leukocytes by increasing the expression of IL-1β and IL-8 at 4 h post-treatment (17).

The purpose of the present study was to evaluate and understand the signaling pathways and immunomodulatory activity of NK-lysin-derived peptides in *S. salar in vitro* and *in vivo* and determine the NK-lysin-derived peptides antibacterial activity against *P. salmonis* (LF-89) and *F. psychrophilum*. Besides, the hemolytic activity of these peptides was evaluated. On the other hand, we reported and characterized two new transcripts of NK-lysin from *S. salar*. In addition, the tissue distribution of three NK-lysins was also established in *S. salar*. The results of this work could provide new insights regarding the antimicrobial and immunomodulatory activities of NK-lysin peptides in salmonids and thus aid in developing a potential alternative for antibiotic use in aquaculture.

## 2 Materials and methods

### 2.1 Sequence analyses

Previously, four putative novel NK-lysin-like peptides from *S. salar* were identified based on the EST database. The four transcripts identified were named *SsNK-lysin 1* (GenBank accession no.: NM_001141110.1), *SsNK-lysin 2* (GenBank accession no.: EG932844.1), *SsNK-lysin 3* (GenBank accession no.: EG810337.1) and *SsNK-lysin 4* (GenBank accession no.: EG819316.1). The present study searched Gene Databases in GenBank (http://www.ncbi.nlm.nih.gov/) for additional putative NK-lysin sequences. All gene and protein sequences were obtained from the National Center for Biotechnology Information (NCBI). The signal peptide cleavage site was predicted with SignalP 5.0 (http://www.cbs.dtu.dk/services/SignalP/). A multiple sequence alignment was performed using the Clustal Omega tool (https://www.ebi.ac.uk/Tools/msa/clustalo/) from the NK-lysin protein sequences of *O. mykiss* and *S. salar*. Besides, another multiple sequence alignment was performed using the ClustalW tool (https://www.genome.jp/tools-bin/clustalw) from NK-lysin protein sequences without signal peptides from mammals, avian, and teleosts. With this last alignment, a phylogenetic tree was constructed with the Molecular Evolutionary Genetic Analysis 11 (MEGA11) program, using the Neighbor-Joining method and the bootstrap test with 1000 replicates. In addition, all ambiguous positions were removed for each pair of sequences (pairwise deletion option), and the Poisson distance correction method was used. The NK-lysin sequences used for phylogenetic analysis and sequence alignment are listed in **Table 1**.

**Table 1:** GenBank accession numbers of the sequences used in the Multiple amino acid alignment and phylogenetic analysis.

Species	Gene name	GenBank No.
*Salmo salar*	NK-lysin 1	NM_001141110.1
*Salmo salar*	NK-lysin 2	XM_014130176.1
*Salmo salar*	NK-lysin 3	EG810337.1
*Salmo salar*	NK-lysin 4	EG819316.1
*Salmo salar*	NK-lysin 5	XM_014125754.1
*Salmo salar*	NK-lysin 6	XM_014129907.1
*Oncorhynchus mykiss*	nkl1 (OmNK1)	LOC110505297
*Oncorhynchus mykiss*	nkl2 (OmNK2)	LOC110498583
*Oncorhynchus mykiss*	nkl3 (OmNK3)	LOC110498133
*Oncorhynchus mykiss*	nkl-like a (OmNKLa)	LOC110498135
*Oncorhynchus mykiss*	nkl-like b transcript variant X1 (OmNKLb X1)	LOC110498134
*Oncorhynchus mykiss*	nkl-like b transcript variant X2 (OmNKLb X2)	XM_036955110.1
*Oncorhynchus mykiss*	nkl-like b transcript variant X3 (OmNKLb X3)	XM_021574736.2
*Oncorhynchus mykiss*	nkl-like b transcript variant X4 (OmNKLb X4)	XM_036955111.1
*Larimichthys crocea*	NK-lysin-like protein (Lcrocea1)	AIL25791.1
*Larimichthys crocea*	NK-lysin-like type 2 protein (Lcrocea2)	ALH22547.1
*Larimichthys crocea*	NK-lysin-like type 3 protein (Lcrocea3)	ALH22548.1
*Danio rerio*	Nkla	KP100115
*Danio rerio*	Nklb	KP100116
*Danio rerio*	Nklc	KP100117
*Danio rerio*	Nkld	KP100118
*Paralichthys olivaceus*	NK-lysin	Hirono et al., 2007 [1]
*Ictalurus punctatus*	NK-lysin type 1 (Ipunctatus1)	NP_001187137
*Ictalurus punctatus*	NK-lysin type 2 (Ipunctatus2)	NP_001187147
*Ictalurus punctatus*	NK-lysin type 3 (Ipunctatus3)	NP_001187232
*Scophthalmus maximus*	NK-lysin	APD51552.1
*Hyporthodus septemfasciatus*	NK-lysin	ALT14560.1
*Cynoglossus semilaevis*	NK-lysin	AGM21637.1
*Fundulus heteroclitus*	NK-lysin (Fheteroclitus1)	JAR79304.1
*Fundulus heteroclitus*	NK-lysin (Fheteroclitus2)	JAR43863.1
*Oreochromis niloticus*	NK-lysin	XP_005477177.1
*Takifugu flavidus*	NK-lysin-like	XP_056867911.1
*Takifugu rubripes*	NK-lysin-like	XP_003962755.1
*Dicentrarchus labrax*	NK-lysin tandem duplicate 4 isoform X1 (DlabraxX1)	XP_051246575.1
*Dicentrarchus labrax*	NK-lysin tandem duplicate 4 isoform X3 (DlabraxX3)	XP_051246595.1
*Lateolabrax japonicus*	NK-lysin	ARS25035.1
*Cyprinus carpio*	NK-lysin (Ccarpio1)	XP_018970060.2
*Cyprinus carpio*	NK-lysin (Ccarpio)	ATD87498.1
*Cyprinus carpio*	NK-lysin tandem duplicate 2 isoform X2 (CcarpioX2)	XP_042598991.1
*Cyprinus carpio*	NK-lysin tandem duplicate 2 isoform X1 (CcarpioX1)	XP_018976518.2
*Sparus aurata*	NK-lysin	QIJ31327.1
*Pangasianodon hypophthalmus*	NK-lysin	UTE79735.1
*Clarias gariepinus*	NK-lysin	QBO59841.1
*Oncorhynchus kisutch*	NK-lysin (Okisutch1)	XP_020356451.1
*Oncorhynchus kisutch*	NK-lysin (Okisutch2)	XP_020343887.1
*Oreochromis aureus*	NK-lysin	XP_031614193.1
*Hippoglossus stenolepis*	NK-lysin	XP_035024225.2
*Sus scrofa*	NK-lysin precursor	NP_001265684.1
*Gallus*	NK-lysin	AMY26518.1
*Homo sapiens*	Granulysin isoform 2 precursor	NP_006424.2

Names in parentheses correspond to those used in phylogenetic analysis and sequence alignment.

### 2.2 Structure analyses and disulfide bonds prediction

SsNK-lysin sequences were modeled using the SWISS-MODEL server (Swiss Bioinformatics Institute, Basel, Switzerland), available at https://swissmodel.expasy.org/interactive (42). A search for templates that could fit the target sequence was performed. From a comprehensive list of more than 100 templates, a heuristic filter was applied to select the 50 most promising models based on coverage and sequence identity criteria. The resulting models were obtained from a variety of sources, including the UniProt database (European Bioinformatics Institute, Cambridge, UK) (43), the Protein Data Bank (PBD) (44), and AlphaFold (DeepMind, London, UK) (44, 45). Subsequently, the models generated for each NK-lysin were subjected to a validation phase, in which tools such as PROCHECK (Laboratory of Molecular Biology, Medical Research Council, Norwich, United Kingdom) (46), VERIFY3D (47), ERRAT (48) and QMEANDisCo (Institute of Bioinformatics, University of Zurich, Zurich, Switzerland) (49) were used. These steps were performed to ensure the integrity and quality of the models obtained.

To predict disulfide bridge formation, the Disulfide by Design 2 server (Boston University School of Medicine, Boston, USA) was used (50). Specific setup parameters included the definition of an angle χ3 with values of -87° or +97°, with a ±30 variation range, and an angle Cα-Cβ-Sγ set at 114.6°, with a tolerance of ±10. The various models generated from the SsNK-lysin sequences were loaded, and the energetic parameters expressed in kcal/mol were evaluated. It is relevant to note that a lower energy reflects a higher probability of disulfide bridge formation. Subsequently, amino acid positions with a higher likelihood of forming disulfide bonds were identified using PyMOL 2.5.5 (DeLano Scientific LLC, San Carlos, USA), and the presence of these bonds with the lowest energy in the analyzed structure was confirmed.

### 2.3 Designing and synthesis of *Salmo salar* NK-lysin-derived peptides

Because the increase in length makes it difficult to obtain NK-lysin by chemical synthesis, short peptides derived from these molecules have been designed and studied. We previously designed and synthesized two small peptides, NK1 and NK2, derived from SsNK-lysin 1 and SsNK-lysin 2, based on alignments between NK-lysin sequences identified in *S. salar* and NKLP27, a peptide derived from *C. semilaevis* NK-lysin (17). NK1 and NK2 comprise 27 residues that form the H2 and H3 α-helices of the SapB domain of SsNK-lysin 1 and SsNK-lysin 2, respectively (17). NK3, NK4, NK5, and NK6 derived from SsNK-lysin 3, 4, 5, and 6 were designed based on sequence alignment between NK-lysin sequences previously identified in *S. salar*. NK3 peptide resulted in 96% identical to NK2, and NK5 and NK6 were identical to NK1. Therefore, we synthesized and characterized three NK-lysin-derived peptides: NK1, NK2, and NK4. The Hydrophobicity, Hydrophobic Moment, total net charge, theoretical isolelectric point, and molecular weights of each peptide were calculated using the Database of Antimicrobial Activity and Structure of Peptides (https://dbaasp.org/home) and Compute pI/Mw from ExPASy (http://web.expasy.org/compute_pi/). The physicochemical properties of the three NK-lysin-derived peptides selected are listed in (**Supplementary Material, Table 1**).

The peptides derived from SsNK-lysin 1, 2, and 4, NK1 (TLKQKLLSVCDKVGFLKSMCKGLMKKH), NK2 (EIKQKLLSYCGKLPLVKSTCEDLVKKH), and NK4 (EIKQKLLSVCDKMGLLKSLCKGMVKKH) were chemically synthesized by GenScript Company (https://www.genscript.com/). Cysteines 10 and 20 were linked by disulfide bonding in the three peptides. The peptides were purified by high-performance liquid chromatography to 90% of purity. Lyophilized peptides were stored at −20°C and dissolved in DMSO before use.

### 2.4 Fish maintenance

Unvaccinated Atlantic salmon (*Salmo salar*) were obtained and maintained in the Marine Biotechnology unit, Faculty of Natural and Oceanography Science, University of Concepcion. The fish occupied in the experiments were certified as free of the most prevalent pathogens. The animals were maintained under a 12: 12 h light: dark cycle and fed daily until satiety with a commercial diet (Micro 200, EWOS).

All the animals used in this study were treated under the Biosecurity Regulations and Ethical Protocols approved by the University of Concepcion Ethics Committee, as required by Chilean Regulatory Entities: National Research and Development Agency (ANID) and National Fisheries and Aquaculture Service (SERNAPESCA).

### 2.5 Antimicrobial assays

The minimal inhibitory concentration (MIC) of synthetic NK-lysin-derived peptides (NK1, NK2, and NK4) was measured for *P. salmonis* (LF89) and *F. psychrophilum* by a broth microdilution method (51). The MIC is defined as the lowest concentration of an antimicrobial agent at which bacterial growth is not detected. *P. salmonis* was grown in Tryptic Soy Broth (TSB) (Merck, Darmstadt, Germany) supplemented with NaCl 3 g/L, fetal bovine serum (FBS) 2.5%, L-cysteine 0.05%, and FeCl3 0.01 g/L (52) and *F. psychrophilum* was grown in a broth containing Tryptone 4 g/L, MgSO_4_ 0.5 g/L, CaCl_2_ 0.5 g/L, Yeast extracts 0.4 g/L, pH 7.2. Briefly, logarithmic phase microorganism cultures were diluted in the broth according to the microorganism to an optical density at 600 nm (OD_600_) of 0.001, approximately equivalent to 10^5^ cfu mL^−1^. Diluted microorganisms (90 μL) were mixed with 10 μL of water (negative control) or peptides in wells of polypropylene microtiter plates (Greiner Bio-One, Germany). The peptides were two-fold serially diluted. The growth was monitored by measuring the change in the absorbance of the culture at 600 nm using a microplate reader after 2 and 5 incubation days at 18 °C for *F. psychrophilum* and *P. salmonis*, respectively. The MIC was determined by visual verification of microbial sedimentation and absorbance reading (600 nm). In addition, the half maximal inhibitory concentration (IC50) was determined, which is a measure of the efficacy of a compound in inhibiting a biological or biochemical function and indicates how much of a given drug or other substance (inhibitor) is needed to inhibit a given biological process by half. The IC50 for each peptide was calculated from concentration-effect curves after non-linear regression analysis using GraphPad prism10 software. MIC and IC50 were expressed as the absolute value of the mean of at least two determinations in triplicate.

### 2.6 Hemolytic assay

The hemolytic activity of NK-lysin-derived peptides (NK1, NK2, and NK4) was determined using human and fish erythrocytes. Briefly, fresh human or fish erythrocytes (5 mL) were washed with PBS and resuspended in PBS (50 mL) supplemented with glucose (0.2%, w/v). Synthetic peptides (serially diluted in PBS) were added to 90 μL of 1% erythrocyte suspension. Samples were incubated for 30 min at 37°C and centrifuged for 10 min at 3500 rpm at room temperature. The supernatants (70 μL) were transferred to a microtiter plate, and the optical density was determined at 405 nm. The percentage of hemolysis was defined relative to the hemolysis obtained with the erythrocyte suspension treated with 0.1% SDS (100% hemolysis). Two individual experiments were performed using duplicate samples for each peptide.

### 2.7 Tissues collection

Five *Salmo salar* weighting 100-150 g were sacrificed by overexposure to benzocaine (20%), and the gill, muscle, intestine, liver, spleen, stomach, head kidney, heart, and skin were aseptically removed for evaluation of constitutive expression of *SsNK-lysin 1, 2* and *4* transcripts. The tissues were kept in RNAlater (Invitrogen) at -80°C until use.

*Salmo salar* weighting 150-200 g were sacrificed by overexposure to benzocaine (20%), and the head kidneys were aseptically removed to isolate head-kidney leukocytes (HKL) for *in vitro* assays.

### 2.8 Isolation of head kidney leukocytes

HKLs were isolated from *S. salar* following the method previously described (53). The head kidney removed aseptically was homogenized through a 40 μm nylon mesh using Leibovitz medium (L-15, Gibco, USA) supplemented with 100 IU/mL penicillin (Gibco, USA), 100 μg/mL streptomycin, 2% heparin and 2% fetal bovine serum (FBS, Hyclone, USA). The resulting cell suspension was placed onto Percoll gradients with a density of 51%/34% and then was centrifuged at 800 g for 40 min at 15°C. The fraction corresponding to the leukocytes was collected, washed twice, and centrifuged at 800 g for 5 min at 15°C in an L-15 medium supplemented with 10% FBS. Viable cell concentration was determined by the Trypan blue exclusion method, and the cells were resuspended in an L-15 medium supplemented with 10% FBS. For each experiment in which HKLs were used, each experimental replicate corresponded to cells from different animals.

### 2.9 Cytotoxicity assay

96-well plates were seeded at a cell density of 500,000 cells per well for HKLs. After 24 hours, the cells were incubated with 50 μM of NK-lysin-derived peptides (NK1, NK2, and NK4) for 24 and 48 hours. After incubation, the cells were incubated with 1 mg/ml MTT at 20°C for 6 hours. Finally, all MTT was removed, and the formazan salts were resuspended in 100 μl isopropanol. Absorbance was then read at 550 nm. Cells without peptides were used as controls for 100% cell viability.

### 2.10 *In vitro* effects on cytokines expression

*S. salar* HKLs were seeded into 24-well culture plates at a concentration of 10^6^ cells/well in L-15 medium with 10% FBS and cultured at 18°C. The cells were incubated with culture media containing 50 μM of the SsNK-lysin-derived peptides (NK1, NK2, and NK4) or culture media alone as negative control. The cells were harvested 6, 12, and 48 hours after the stimulation. For all treatments, cells were kept in incubation at 18°C. Expression of TNF-α, IL-1β, IL-8, IFN-γ, IL-10, IL-18, Mx, and TGF-β was determined by qRT-PCR.

### 2.11 Inhibitor assay

HKLs (4x10^6^ cells/well) were pretreated for 2 hours with 10 μM of SB202190 inhibitor (p38 inhibitor) (Sigma Aldrich), U0126 inhibitor (mitogen-activated protein kinase 1/2 (MEK1/MEK2) inhibitor) or SP600125 inhibitor (JNK inhibitor) (Abcam). As a negative control, cells were incubated with 0.1% DMSO as a vehicle for 2 hours. Cells were then incubated with 50 μM of NK1, NK2, NK4, or medium (negative control) for 12 hours. For all treatments, cells were kept in incubation at 18°C. Cell lysates were harvested, RNA was extracted, and IL-1β relative expression was analyzed by qRT-PCR.

### 2.12 PAMP induced response

SHK-1 cells were seeded in L-15 medium (supplemented with 10% FBS) at 3x10^6^ cells/well. After 24 hours, the cells were co-stimulated with 50 μM of SsNK-lysin-derived peptides (NK1, NK2, and NK4) and 1 µg/mL of lipopolysaccharide (LPS from *E. coli*, 0111:B4) (Sigma Aldrich) or 1 µg/mL of poly(I:C) (Sigma Aldrich) for 6 and 12 hours. Besides, cells were treated only with LPS, poly(I:C), or peptides. Negative control cells with culture media alone were included. For all treatments, cells were kept in incubation at 18°C. Expression of TNF-α, IL-1β, and IL-8 as an LPS-induced response was determined by qRT-PCR. For poly(I:C), the expression of IFN-1α and Mx involved in the antiviral response was determined by qRT-PCR.

### 2.13 *In vivo* effects on cytokines expression

Twenty-five *S. salar* per group of approximately 50 g of body weight will be acclimatized for two weeks at 10-12°C. After this period, animals were intraperitoneally injected with SsNK-lysin-derived peptides (NK1, NK2, and NK4) (20 μg per fish). At 1-, 3-, 7-, 14- and 21 days post-injection, fish (n = 5 per treatment group per time point) were euthanized, and head kidneys were collected. Immediately after tissue extraction, tissues were stored in RNAlater (Thermo Fisher Scientific) at -80°C until use. Expression of IL-1β, IL-8, IFN-γ, Mx, IL-4/13 and IL-22 was determined by qRT-PCR.

### 2.14 RNA extraction

RNA extraction from tissue samples, SHK-1 cells, and HKLs was performed using TRIzol reagent (Thermo Fisher Scientific) according to the manufacturer’s instructions. Tissue samples were homogenized in TRIzol reagent before RNA extraction. For all assays, RNA concentration, and purity were assessed using a Sinergy^®^ HTK Take3 microplate reader (BioTek, Agilent Technologies), and RNA integrity was verified by 1% agarose gel electrophoresis. RNA samples were stored at -80°C until use.

### 2.15 Reverse transcription

For the analysis of tissue expression of SsNK-lysin transcripts, a pool was prepared from each tissue (5 samples each). The samples were treated with DNAse I # M0303 (New England Bio Labs), using 2 μg of RNA in a final volume of 11 μL, according to the manufacturer’s protocols. Reverse transcription was performed using the RevertAid First Strand cDNA Synthesis Kit (Thermo Fisher Scientific) according to the following protocol: 11 μL of DNAse I treatment reaction, 1 μL of random hexamer primer, 4 μL of 5X reaction buffer, 2 μL of dNTP mix (10 mM), 1 μL of Ribolock RNase inhibitor (20 U/μL), 1 μL of RevertAid RT (200 U/μl) in a final volume of 20 μL. The reactions were incubated in the TProfessional Basic Thermocycler (Biometra) at 25°C for 5 minutes, then at 42°C for 60 minutes, and finally at 70°C for 5 minutes. A negative control without retrotranscriptase (No-RT control) was performed for each tissue.

### 2.16 RT-qPCR protocols

The Primer-BLAST tool (https://www.ncbi.nlm.nih.gov/tools/primer-blast/) was used for primer design. Specific oligonucleotides, which did not amplify transcript variants and were aligned with an exon-exon junction (not applicable for predicted sequences), were selected for the desired products. Sequences and information for the primers used in all RT-qPCR assays are shown in **Table 2**.

**Table 2:** Primer sequences used in qRT-PCR assays.

Gene name		Sequence 5’-3’	Amplicon size (bp)	NCBI access number	Amount of RNA/cDNA (ng)*
SsNK-lysin 1	Forward	ATTGCAGTACATTTTGTATCATCTCCAAATG	119	NM_001141110.1	100
	Reverse	TGAGCTTTATTTTTTAGCTAGCC			
SsNK-lysin 2	Forward	GTGTCAGTCTTAGTCTTAAACTG	144	XM_014130176.1	100
	Reverse	TACTATCAATTGAGGTTTATTTTTTGC			
SsNK-lysin 4	Forward	GTGTATAGTCATTCTTAAATTGCAGT	115	XM_014210204.2	100
	Reverse	TTCACACAAACATAAAACATTCG			
SsIL-10	Forward	CGCTATGGACAGCATCCTGAAGTTC	118	XM_014168417.1	20
	Reverse	GTGGAAGATGTTTCCGATGGAGTCG			
OmIL-1β	Forward	ACATTGCCAACCTCATCATCG	91	AJ223954	20
	Reverse	TTGAGCAGGTCCTTGTCCTTG			
SsIL-8	Forward	GGCCCTCCTGACCATTACT	102	NM_001140710	20
	Reverse	ATGAGTCTACCAATTCGTCTGC			
SsIL-18	Forward	AGCAGATGATTGCCGGTTCA	129	NM_001141408.1	200
	Reverse	TTCTTCTCGCAGCACACCAT			
SsTGF-β	Forward	GGCCATCCGTGGACAGATAC	92	XM_014196504.1	20
	Reverse	GGGAGGTTGGGACTTTCTCG			
SsIFN-γ	Forward	CCGTACACCGATTGAGGACT	133	FJ263446.1	200
	Reverse	GCGGCATTACTCCATCCTAA			
TNF-α1	Forward	TGTGTGGCGTCCTCTTAGTAGCAGCTT	101	NM_001123589.1	200
	Reverse	CTCCATTTTGTCCTGCATCGTTGC			
SsIL-4/13a	Forward	ACCACCACAAAATGCAAGGAGT	70	NM_001204895.1	200
	Reverse	ACGTGGCATTTTTCACGGAG			
OmIL-22	Forward	ATCTGCTGCCTGCATGCTAA	151	NM_001164064.1	200
	Reverse	TAGCACAGCCGTGTTCCTTC			
SsMx	Forward	TCGGGAAATGGAAGGCACAA	99	XM_014133087.2	20
	Reverse	CCCTTCCACGGTACGTCTTC			
SsIFN-1α	Forward	CCTGCCATGAAACCTGAGAAGA	108	NM_001123710.1	20
	Reverse	TTTCCTGATGAGCTCCCATGC			
SsEF-1α	Forward	CACCACCGGCCATCTGATCTACAA	78	AF321836	20
	Reverse	TCAGCAGCCTCCTTCTCGAACTTC			

Ss correspond to Salmo salar.

Om correspond to Oncorhynchus mykiss.

*Amount of RNA/cDNA that was used per RT-qPCR reaction.

RT-qPCR reactions were performed with cDNA samples to analyze the expression of *SsNK-lysin 1, 2*, and *4*. The KAPA SYBR FAST One-Step qRT-PCR Master Mix (Kapa Biosystems, USA) was used according to the following protocol: 5 μL of qPCR Master Mix (2X), 0.2 μL of forward primer (10 μM), 0.2 μL of reverse primer (10 μM), 1 μL of cDNA and 3.6 μL of water, in 10 μL final volume. The thermal profile for all genes was as follows: 90°C for 3 minutes, 90°C for 10 seconds (40 cycles), and 58°C for 20 seconds. A no reverse transcriptase (No-RT) control was included for each tissue, and a no cDNA control (No Template Control, NTC) was included for each gene. Each sample measurement was repeated three times.

RT-qPCR reactions were performed on RNA samples for inhibitor treatment, PAMPs-induced response, and *in vitro* and *in vivo* stimulation with SsNK-lysin-derived peptides. In all cases, the Brilliant II SYBR^®^ Green qRT-PCR Master Mix, 1-Step kit (Agilent, USA) was used according to the following protocol: 2 μL of RNA (amounts described in **Table 2**), 0.4 μL of RT/RNase block enzyme mixture, 5 μL of 2X Brilliant II SYBR Green qRT-PCR Master Mix, 2.36 μL of water, 0.24 μL of mix forward and reverse primers (2.5 μM each) in 10 μL final volume. The cycling conditions for all genes and assays were as follows: 50°C for 30 minutes (RT reaction), 95°C for 10 minutes, 95°C for 15 seconds, and 58°C for 30 seconds (40 cycles). No RNA controls (NTC) were included for all genes and assays, and each sample measurement was repeated three times.

The EF-1α reference gene was used as a normalizer for all RT-qPCR assays. For the tissue SsNK-lysin expression assay, stomach tissue was used as a control or calibrator. For the other assays, untreated cells or tissues from untreated animals were used as calibrators. The AriaMx real-time PCR system (Agilent, USA) was used for all RT-qPCR reactions. In addition, to standardize and validate the primers used and the reactions, dynamic ranges, efficiency calculation, melting curve evaluation, and visualization of amplicons in 1% agarose gel electrophoresis were performed. The results were analyzed using the comparative Ct (2^-ΔΔCt^) method (54), and GraphPad Prism 10 software was used for graph generation and statistical analysis. Specifically for constitutive tissue expression, relative expression was determined according to Paff’s mathematical model (2001) (55).

### 2.17 Phagocytosis assays

Phagocytic activity was analyzed by a microplate fluorometric assay using pHrodoTM Green *E. coli* Bioparticles™ conjugate (Molecular Probes/Thermo Fisher Scientific). Particles were suspended in Live Cell Imaging Solution buffer (Molecular Probes/Thermo Fisher Scientific) supplemented with 0.2% (w/v) glucose (LCIS-glu) at a density of 1 mg/mL, vortexed for 2 minutes and sonicated for 5 minutes at room temperature. HKLs were seeded at 1x10^6^ cells/well density in 96-well plates in L-15 medium supplemented with 2% FBS and incubated at 18°C for 24 hours. Cells were washed with HBSS, and the phagocyte-enriched population was incubated for 30 minutes at 18°C with 50 μL of LCIS-Glu and then with 50 μL of bioparticles. SsNK-lysin-derived peptides (NK1, NK2, and NK4) were evaluated at 0, 10, and 50 μM. Control wells without cells containing bioparticles were included in each plate to allow subtraction of background fluorescence from the particles at neutral pH. Cells plus peptide and bioparticles were incubated for 3 hours at 18°C. Fluorescence was recorded on the Synergy HTX microplate reader (BioTek Instruments, USA) in well area scanning mode, with excitation and emission wavelengths of 488 and 528 nm, respectively. Data were calculated as percentage phagocytosis relative to control cell samples incubated with bioparticles without stimuli after subtracting background particle fluorescence (cell-free control). Each assay was performed in triplicate and repeated with at least two independent samples.

### 2.18 Statistical analysis

All data were assessed for homogeneity of variances and normal distribution before data were analyzed by one-way ANOVA. The Dunnett *post hoc* test compared means from experimental groups against a control group mean. Šidák’s multiple comparisons test was used to perform simultaneous joint pairwise comparisons for all possible pairwise combinations of means. Statistical analysis was done using GraphPad PRISM version 10.00 (GraphPad Software, San Diego, CA). *p*<0.05 was considered a significant difference.

## 3 Results

### 3.1 Sequence and structure analyses

Based on the Gene database, this study identified two additional putative novel NK-lysin-like peptides from *Salmo salar*. The two transcripts identified were named *SsNK-lysin 5* (GenBank accession nos.: XM_014125754.1) and *Ss-NK-lysin 6* (GenBank accession nos.: XM_014129907.1). Besides, we identified the genes for the six SsNK-lysin-like peptides (**Table 3**). *SsNK-lysin 1, 2, 3, 5*, and *6* are located on chromosome 1, whereas the gene for *SsNK-lysin 4* is located on chromosome 9. **Table 3** also shows the orientation of these genes on chromosome arms 1q and 9q of the *Salmo salar* genome.

**Table 3:** Characteristics of NK-lysin genes from *Salmo salar*.

Protein	Genomic DNA^a^	Location	Orientation	cDNA^b^	Full length (aa)	Signal peptide/mature peptide (aa)	Theoretical pI/Mw^c^ of mature peptide
SsNK-lysin 1	AGKD04000266.1	1,375,167-1,376,740	R	NM_001141110.1	127	22/105	6.76/12041.04
SsNK-lysin 2	AGKD04000266.1	1,558,970-1,560,152	R	XM_014130176.2	129	22/107	8.67/12200.33
SsNK-lysin 3	JAIUJH010000113.1	7,028,490-7,029,669	R	EG810337.1	129	22/107	9.10/12208.42
SsNK-lysin 4	CAKNVC010002408.1	15,493,260-15,494,388	F	XM_014210204.2	136	22/114	8.18/12992.35
SsNK-lysin 5	CAJNNT020000177.1	34,778,852-34,780,133	R	XM_014125754.2	133	22/111	8.18/12629.84
SsNK-lysin 6	CAKNVA020000301.1	34,368,232-34,369,249	F	XM_014129907.2	133	22/111	8.14/12615.85

a Accession number of whole-genome shotgun counting (wgs).

b Accession number of cDNA.

c Theoretical pI (isoelectric point) and Mw (molecular weight).

The *SsNK-lysin 5* open reading frames (ORF) consisted of 402 bp and encoded a protein of 133 amino acid residues. It contains an N-terminal signal peptide (1–22 aa) and a SapB domain (49–123 aa). The theoretical molecular mass of the mature SsNK-lysin 5 is 12629.84 Da, and the isoelectric point (pI) is 8.18 (**Table 3**). The *SsNK-lysin 6* ORF consisted of 402 bp and encoded a protein of 133 amino acid residues. It contains an N-terminal signal peptide (1–22 aa) and a SapB domain (49–123 aa). The theoretical molecular mass of the mature SsNK-lysin 6 is 12615.85 Da, and the pI is 8.14 (**Table 3**).

**Figure 1:**
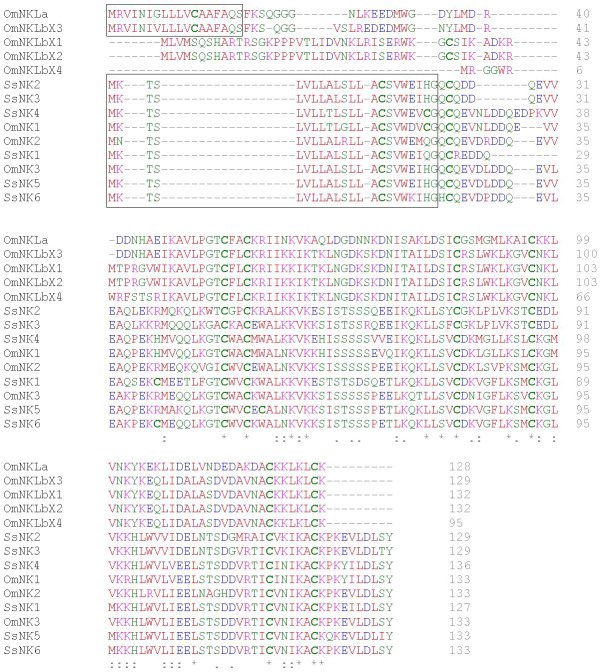
Multiple alignment of salmonid NK-lysin sequences. Multiple alignment of NK-lysin sequences from *S. salar* and *O. mykiss* was performed with the Clustal Omega tool. The sequences corresponding to the signal peptide predicted with SignalP 5.0 are framed. The physicochemical properties of the amino acid residues are represented by different colors, blue: acidic; red: small ]small+ hydrophobic (incl.aromatic -Y)]; magenta: basic and green: hydroxyl + sulfhydryl + amine + G. In addition, “*” indicates positions with a single fully conserved residue, “:” indicates conservation between residues with highly similar properties, and “.” indicates conservation between residues with weakly similar properties. Cysteine residues are highlighted in bold.

The SsNK-lysin 5 encoded protein sequence shared 84.3%, 74.2%, 75%, and 77.4% identity with the Ss-NK-lysin 1, 2, 3, and 4 (reported in 2019), respectively (17). The SsNK-lysin 6 encoded protein sequence shared 86.6%, 71.9%, 72.7%, and 76.7% identity with the Ss-NK-lysin 1, 2, 3 and 4, respectively (17). Besides, the SsNK-lysin 5 and SsNK-lysin 6 encoded protein sequences shared 90.98% identity. On the other hand, the percent of the identity of SsNK-lysin peptides with NK-lysin (NKL) peptides from *O. mykiss* (determined without signal peptide) ranged from 71% to 85% (22). However, the percent of the identity of SsNK-lysin peptides with Nkl-like a and four variants of Nkl-like b from *O. mykiss* ranged from 27% to 31% (22) (**Figure 1**).

**Figure 2:**
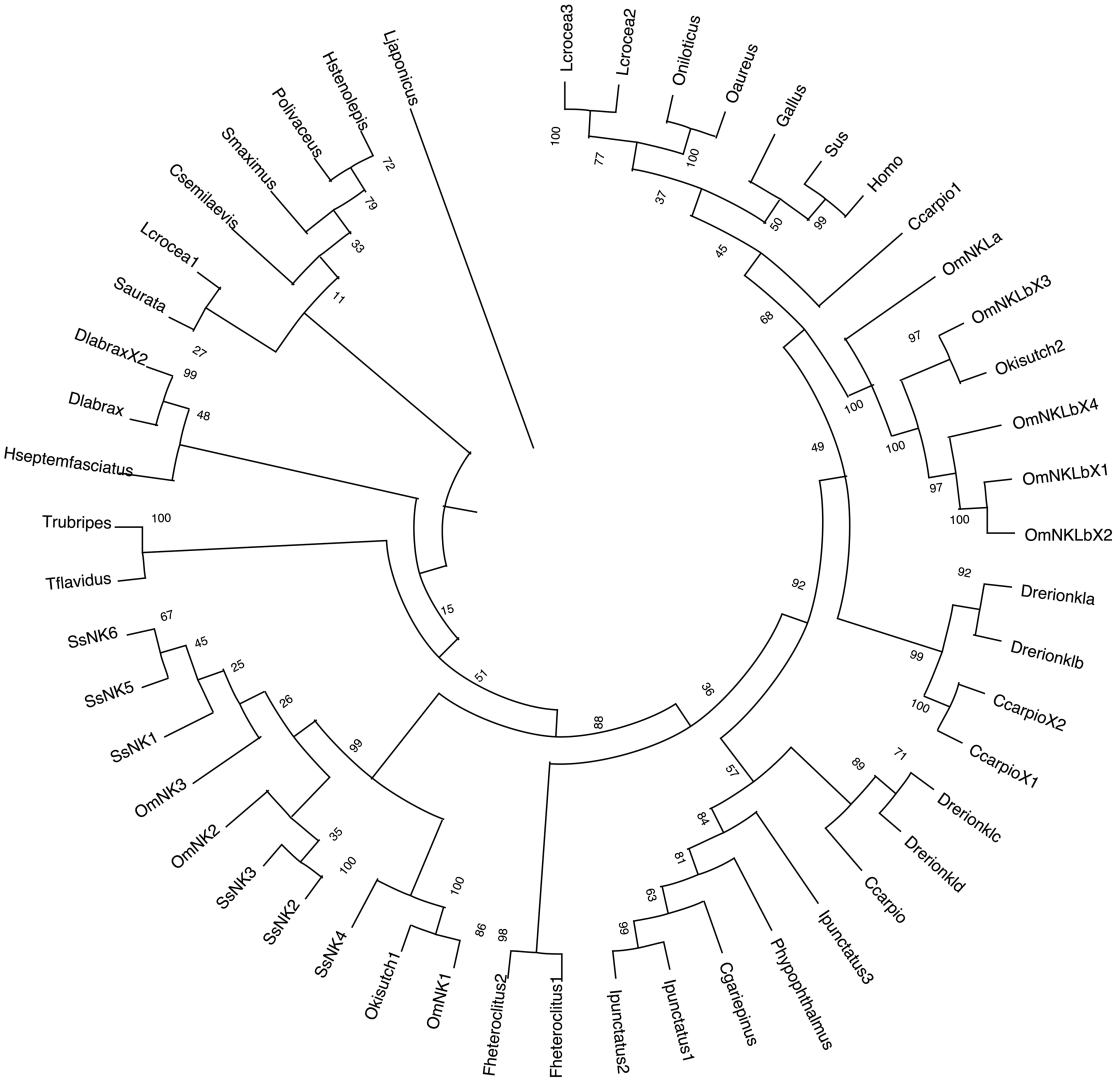
Phylogenetic analysis of NK-lysin amino acid sequences. A phylogenetic tree was constructed using the Neighbor-joining method. Numbers above the branches indicate frequencies per 1000 Bootstrap analysis. NK-lysin protein sequences without signal peptides from mammals, birds, and teleosts were used for alignment and phylogenetic tree construction. NCBI GenBank accession numbers of sequences used are listed in **Table 1**.

The inferred phylogenetic tree showed that the mammalian and avian NK-lysin sequences cluster within the clade furthest from the rest of the phylogenetic tree and are related to the NK-lysin sequences of the *L. crocea* species and the evaluated sequences of the *Oreochromis* genus. On the other hand, all *S. salar* sequences and most NK-lysin sequences of other salmonids are grouped in the same clade and subdivided into sub-branches. One sub-branch groups the SsNK-lysin 4 sequence with those of other salmonids, suggesting that this sequence may be less related to the other *S. salar* sequences. On the other hand, SsNK-lysin 2 and SsNK-lysin 3 cluster in another sub-branch closer to OmNK2, while SsNK-lysin 1, SsNK-lysin 5 and SsNK-lysin 6 cluster together with OmNK3 in another sub-branch of this clade. The rest of the salmonid NK-lysin sequences, including the NK-lysin-like sequences from *O. mykiss* and its variants, cluster in a distant clade, closer to sequences from species such as *C. carpio*, *L. crocea*, *O. niloticus*, *O. aureus*, and avian and mammalian sequences (**Figure 2**).

Multiple alignments revealed six cysteine residues in the two SsNK-lysin mature peptides reported here that are highly conserved among fish species. Nevertheless, all SsNK-lysins contain at least an additional cysteine residue conserved among them (**Figure 1**). SsNK-lysin 1 and SsNK-lysin 6 have eight cysteine residues in the mature peptide conserved among them, and SsNK-lysin 5 possesses eight cysteine residues; one of them is not conserved among SsNK-lysin sequences.

**Figure 3:**
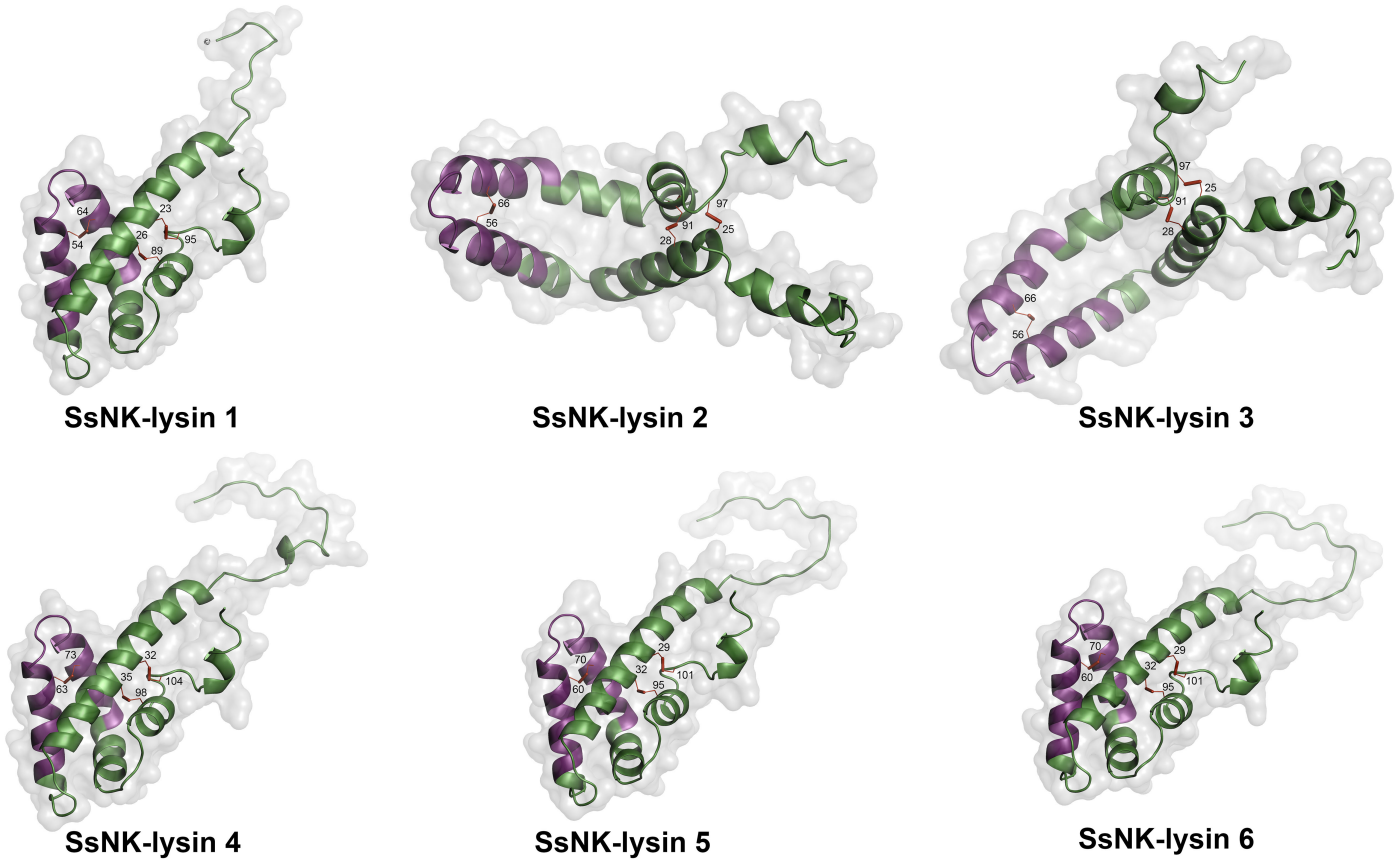
Modeled structure of *Salmo salar* NK-lysin peptides (SWISS‐MODEL server). The region corresponding to the NK-lysin-derived peptides is indicated in magenta. The cysteines involved in the disulfide bonds are also indicated.

To predict the structures and the formation of disulfide bridges, we used the SWISS-MODEL server (Swiss Bioinformatics Institute, Basel, Switzerland) (42) and the Disulfide by Design 2 server (Boston University School of Medicine, Boston, USA) (50), respectively. As a result, we found that three disulfide bridges are predicted to be generated for all sequences, with one or two cysteine residues not forming bridges. The positions of the bridging cysteines are shown in **Table 4**. **Figure 3** shows the structures corresponding to the SsNK-lysin. Visualization of the protein revealed a predominance of α-helices, which are extensively distributed along the molecule. No folded β-sheets were detected in the current protein conformation. Additionally, segments known as loops or turns were identified. These segments could act as flexible connections facilitating continuity between ordered structures. Loops and unstructured areas indicate sites of flexibility and movement capability within the protein.

**Table 4:** *Salmo salar* NK-lysins disulfide bridges predicted by Disulfide by Design 2 server.

ID	Template (AlphaFold)	Sequence identity (%)	Specie	Cys 1	Cys 2	Energy (kcal/mol)
SsNK-lysin 1	A0A060YEW6	78.10	*Oncorhynchus mykiss*	23	95	0.24
				26	89	0.48
				54	64	0.50
SsNK-lysin 2	A0A4W5LUK4	74.29	*Hucho hucho*	25	97	0.32
				28	91	0.44
				56	66	1.22
SsNK-lysin 3	A0A4W5LUK5	75.24	*Hucho hucho*	25	97	0.28
				28	91	0.41
				56	66	1.22
SsNK-Lysin 4	A0A060YEW6	77.48	*Oncorhynchus mykiss*	32	104	0.25
				35	98	0.28
				63	73	0.51
SsNK-lysin 5	A0A060YEW6	84.68	*Oncorhynchus mykiss*	29	101	0.24
				60	70	0.49
				32	95	0.53
SsNK-Lysin 6	A0A060YEW6	84.68	*Oncorhynchus mykiss*	29	101	0.21
				60	70	0.48
				32	95	0.57

### 3.2 Antimicrobial activity of NK-lysin-derived peptides

The antimicrobial activity of peptides derived from SsNK-lysin against *P. salmonis* and *F. psychrophilum* was determined. Bacteria were chosen to represent important fish pathogens. Results show that NK1 and NK4 are active against *P. salmonis*. In contrast, the three peptides were active against *F. psychrophilum*. SsNK-lysin-derived peptides over a range of concentrations up to 200 μM displayed variable degrees of antimicrobial activity against the microorganisms tested (**Table 5**). NK1 and NK4 were the most active peptides against these critical fish pathogens. In contrast, NK2 exhibited a more limited spectrum of activity against these pathogens.

**Table 5:** Antimicrobial spectrum of synthetic NK-lysin derived peptides isolated from *Salmo salar*.

Peptide name	Microorganisms	MIC^a^ (μM)	IC50^b^ (μM)
NK1	*Pisciricketsia salmonis*	6.25	1.3
	*Flavobacterium psychrophilum*	12.5	2.02
NK2	*Pisciricketsia salmonis*	>200 µM	_
	*Flavobacterium psychrophilum*	100	35
NK4	*Pisciricketsia salmonis*	12.5	5.03
	*Flavobacterium psychrophilum*	50	6.3

a MIC: Minimal inhibitory concentration.

b IC50: The half maximal inhibitory concentration.

### 3.3 Hemolytic activity

**Figure 4:**
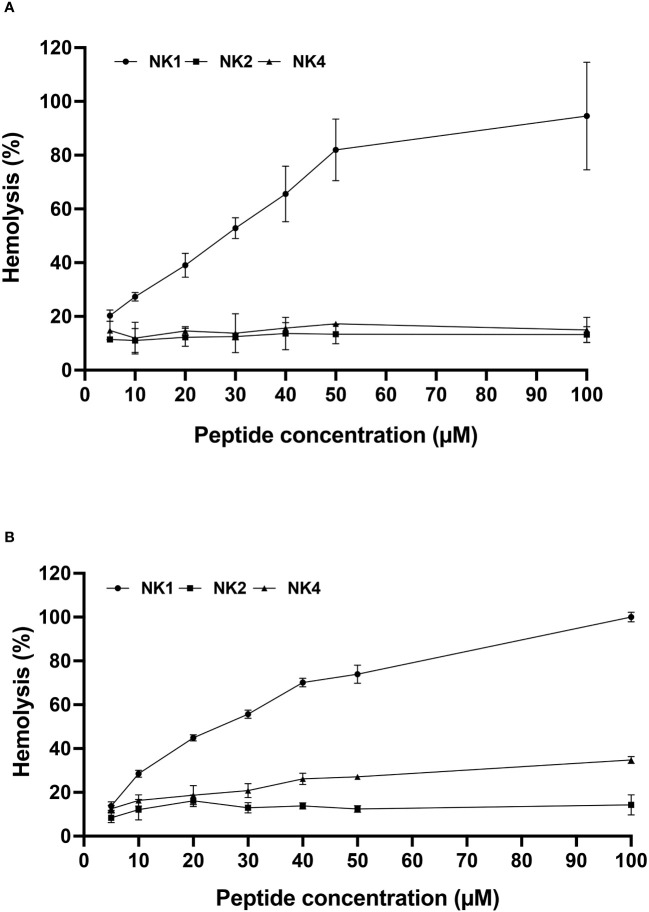
Hemolytic activity of the three *Salmo salar* NK-lysin-derived peptides against fish **(A)** and human **(B)** erythrocytes. The percentage of hemolysis was defined relative to the hemolysis obtained with the erythrocyte suspension treated with 0.1% SDS (100% hemolysis).

Synthetic SsNK-lysin-derived peptides were assays for hemolytic activity in human and fish red blood cells. NK2 and NK4 were not hemolytic for fish red blood cells at concentrations below 100 μM. In contrast, NK1 was hemolytic in fish erythrocytes, reaching 50% of hemolysis at approximately 30 μM (**Figure 4A**). In addition, hemolytic activity in human red blood cells was observed for NK1 in a dose-dependent manner, reaching 50% of hemolysis at 25 μM approximately. On the other hand, NK4 reached 33% of hemolysis at the highest concentration analyzed (**Figure 4B**). In contrast, NK2 was not hemolytic for human erythrocytes at concentrations below 100 μM (**Figure 4B**).

### 3.4 NK-lysins tissue-distribution pattern

We analyzed the constitutive expression of the three *Salmo salar* NK-lysin transcripts, *SsNK-lysin 1*, *SsNK-lysin 2*, and *SsNK-lysin 4*. The high sequence homology of the salmon NK-lysin transcripts does not allow us to design specific oligonucleotides for NK-lysin variants 3, 5, and 6.

**Figure 5:**
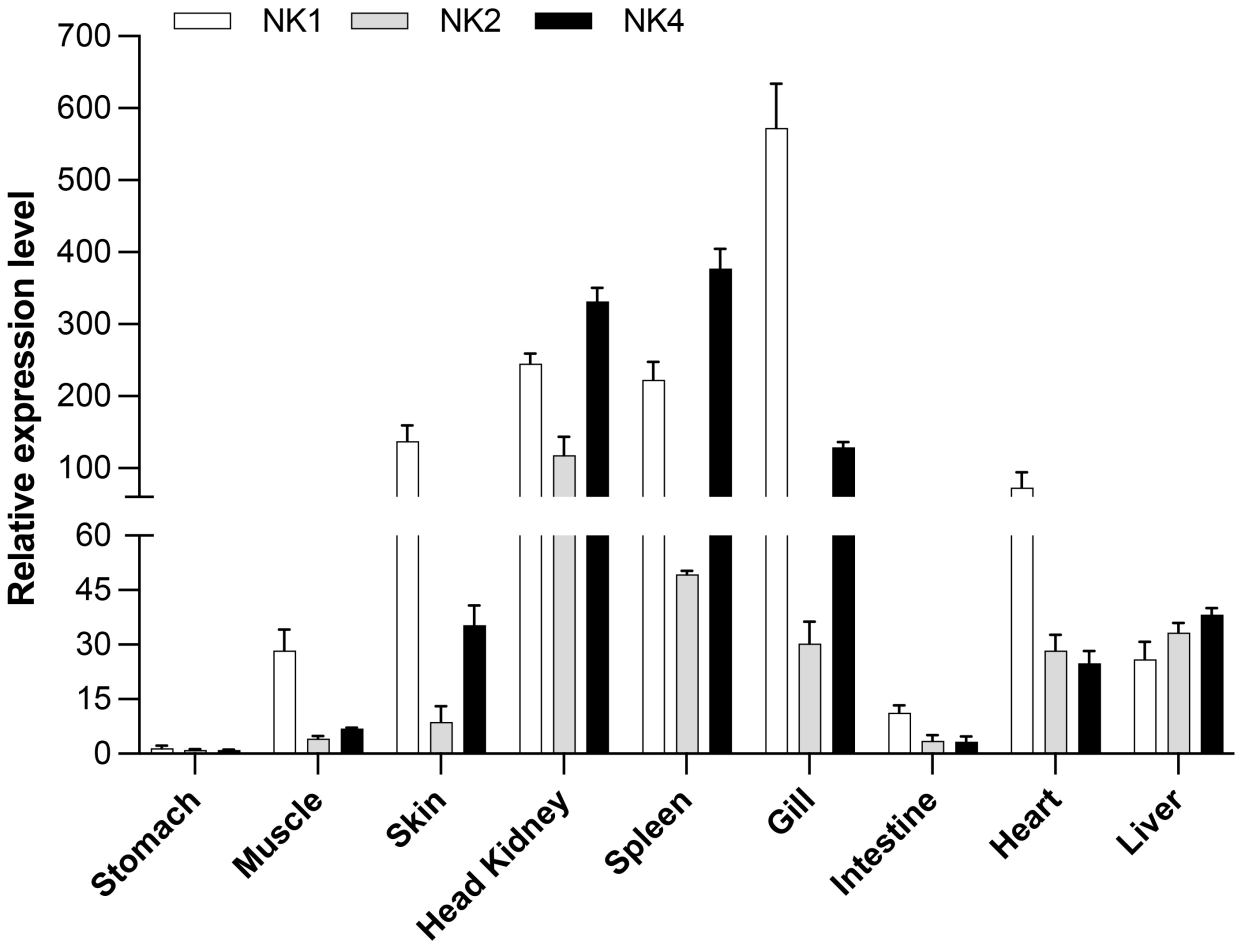
Relative SsNK-lysin 1, 2, and 4 mRNA expression profiles in nine uninfected Atlantic salmon (*S. salar*) tissues. The EF-1α reference gene was used as a normalizer, and the stomach tissue was used as a control or calibrator. The relative expression was determined according to Paff’s mathematical model (2001) (55). The data was shown as mean ± SD (n=5).

When the constitutive expression of *SsNK-lysin 1, 2*, and *4* were analyzed in different *S. salar* tissues, different expression levels were observed for each transcript analyzed (**Figure 5**). *SsNK-lysin 1* was highly expressed in skin, head kidney, spleen, and gills, showing its highest expression level in gills. Besides, *SsNK-lysin 1* was also expressed in muscle, intestine, liver, and heart; *SsNK-lysin 2* was expressed in all tissues analyzed but showed a lower expression level than the other two transcripts analyzed. On the other hand, *SsNK-lysin 4* showed its highest expression level in the gills, spleen, and head kidney. In addition, *SsNK-lysin 4* was also expressed in muscle, skin, liver, heart, and intestine. The intestine showed the lowest expression level for all transcripts analyzed.

### 3.5 Cytotoxicity toward head kidney leucocytes

**Figure 6:**
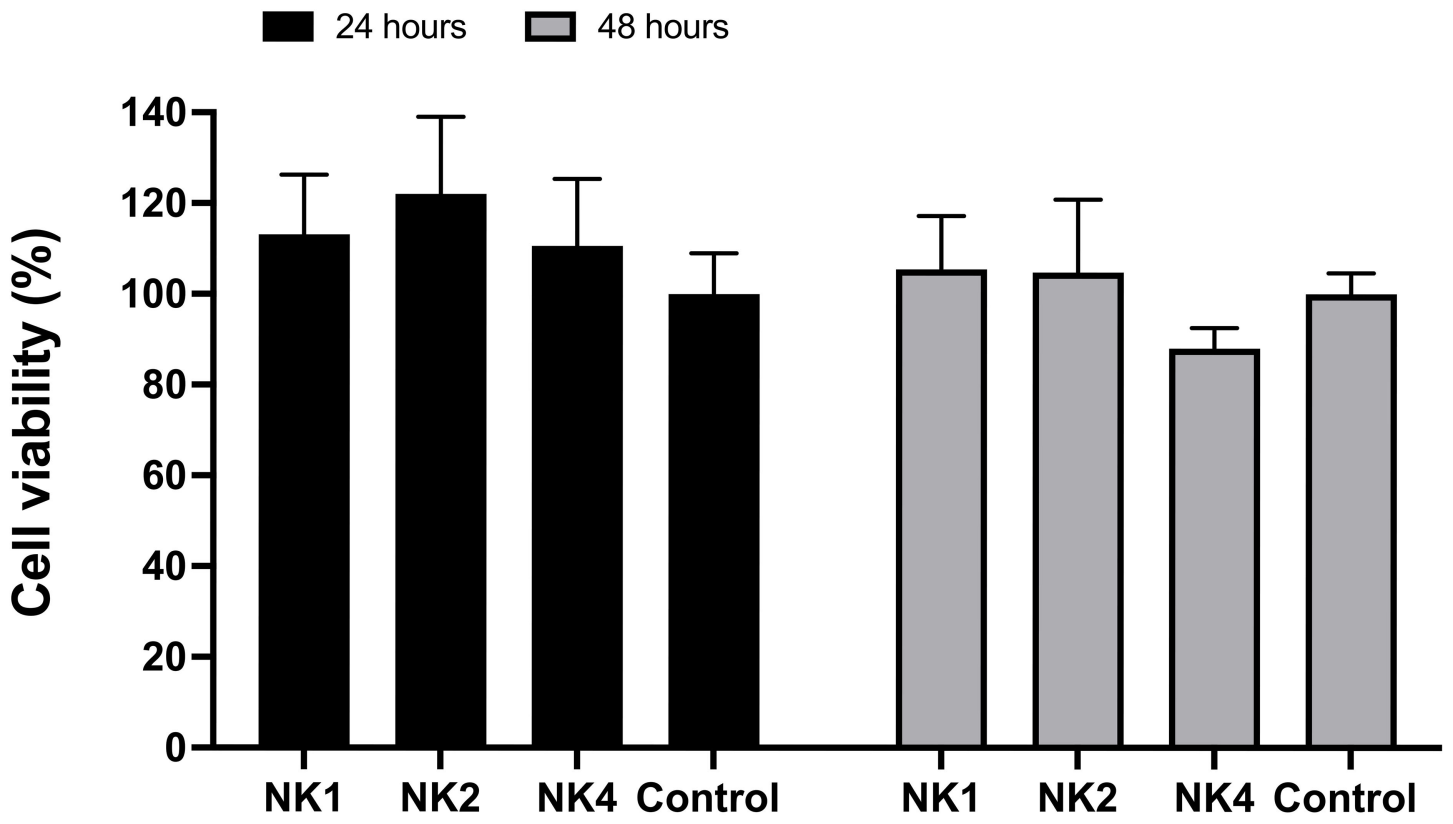
Effect of NK-lysin-derived peptides (NK1, NK2, and NK4) on cell viability (MTT assay) in head kidney leucocytes from *Salmo salar* after 24 and 48 hours of treatment with peptides at a concentration of 50 μM. Values are expressed as means ± S.D. (n=6).

The cytotoxicity of the NK-lysin-derived peptides on *S. salar* HKLs was assessed by a standard MTT assay conventionally used to measure cell viability. As shown in **Figure 6**, none of the peptides were significantly toxic to *S. salar* HKLs at 50 μM, at 24 and 48 hours of treatment, with cell viability greater than 87% at this concentration.

### 3.6 Cytokine expression induced by SsNK-lysin-derived peptides in *Salmo salar* head kidney leucocytes

**Figure 7:**
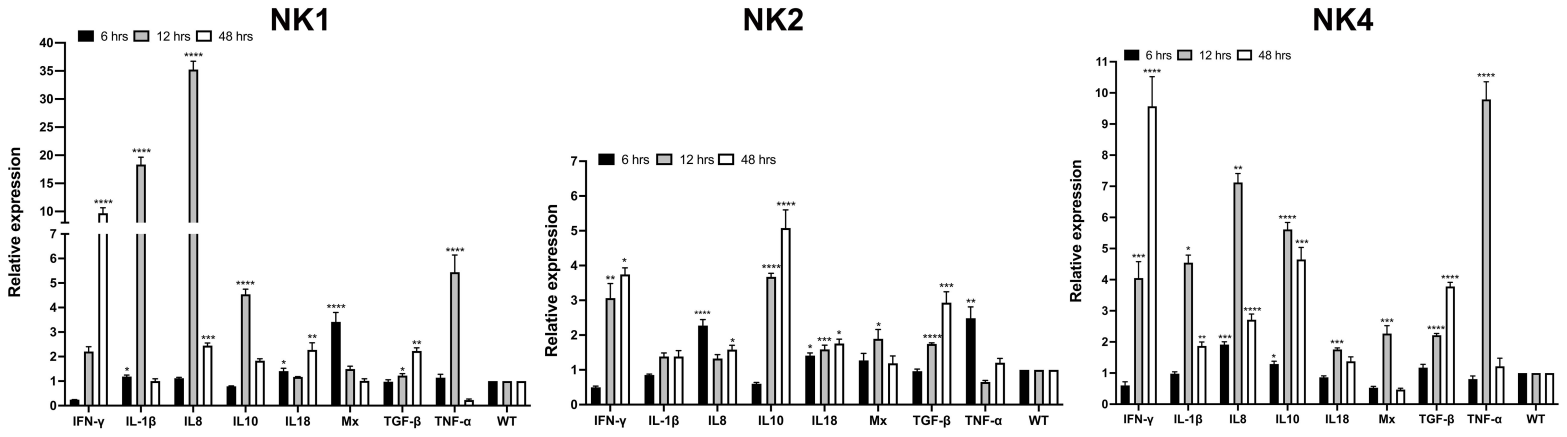
Relative expression of immune-related genes induced by SsNK-lysin-derived peptides in *S. salar* head kidney leucocytes. Cells were stimulated with 50 μM of the synthetic NK-lysin-derived peptides for 6, 12, and 48 h. Expression levels were analyzed by Real-time PCR. The expression of the mRNA was analyzed as 2^−ΔΔCT^ relative quantification. The comparative threshold cycle values were normalized for EF-1α. The comparative threshold cycle values were normalized for EF-1α. Data were expressed as the means ± S.D. of three independent experiments, each in triplicate. Data were analyzed by ANOVA followed by Dunnett’s multiple comparisons test (* p<0.05; ** p<0.01; ***p < 0.001; **** p<0.0001). The Dunnett *post hoc* test was used to compare means from experimental groups against a control group mean.

The ability of SsNK-lysin-derived peptides (NK1, NK2, and NK4) to induce cytokine expression in the *S. salar* HKL was evaluated by RT-qPCR. The mRNA expression of IL-10, TGF-β, TNF-α, IL-8, IL-1β, Mx-1, IFN-γ and IL-18 was evaluated after 6, 12, and 48 h post-treatment with synthetic peptides. NK1 at 50 μM induced a significant increase in the expression of pro-inflammatory cytokines IL-1β (6- and 12-hours; 1.2- and 18.3-fold change, respectively), IL-8 (12- and 48-hours; 35- and 2.4-fold change, respectively), IL-18 (6- and 48-hours; 1.4- and 2.3-fold change, respectively) and TNF-α (12 hours; 5.4 folds respect to control). Besides, NK1 at 50 μM induced a significant increase in the expression of T_H_1 cytokine IFN-γ at 48 hours post-treatment, reaching 9.6-fold concerning control. NK1 also induced a significant increase in the expression of IFN-γ inducible cytosolic protein Mx-1 at 6 hours (3.4-fold change). Respect to anti-inflammatory cytokines, NK1 at 12 hours post-treatment induced a significant increase in the expression of IL-10 (4.5-fold change) and induced a significant increase in the expression of TGF-β at 12- and 48-hours post-treatment (1.2- and 2.2- fold change, respectively) (**Figure 7**).

On the other hand, NK2 at 50 μM induced a significant increase in the expression of pro-inflammatory cytokines IL-8 (6- and 48-hours; 2.3- and 1.6-fold change, respectively) and IL-18 (6-, 12- and 48-hours; 1.4-, 1.6- and 1.8-fold change, respectively) and TNF-α (6 hours; 2.5-folds respect to control) (**Figure 7**). Besides, NK2 at 50 μM induced a significant increase in the expression of IFN-γ at 12- and 48-hours post-treatment reaching 3- and 3.7-folds respect to control, respectively. NK2 also induced a significant increase in the expression of Mx-1 at 12 hours (1.9-fold change). Concerning anti-inflammatory cytokines, NK2 at 12- and 48-hours post-treatment caused a significant increase in the expression of IL-10 (3.7- and 5.1-fold change, respectively) and TGF-β (1.7- and 2.9-fold change, respectively) (**Figure 7**).

NK4 at a dose of 50 μM induced the expression of all cytokines analyzed. At 6 hours post-treatment, NK4 induced the expression of IL-8 and IL-10, reaching 1.9- and 1.3-fold concerning control, respectively. The higher expression levels were observed for IFN-γ at 12- and 48-hours, IL-1β at 12 hours, IL-8 at 12 hours, IL-10 at 12 hours, and TNF-α at 12 hours (4-, 9.5-, 4.5-, 7.1-, 5.6- and 9.8- fold change, respectively). Besides, NK4 induced a significant increase in the expression of IL-1β, IL-8, IL-10 and TGF-β at 48 hours post-treatment (1.8-, 2.7-, 4.6- and 3.7- fold change, respectively) (**Figure 7**).

### 3.7 Role of MAPK signaling pathways in the IL-1β expression induced by SsNK-lysin-derived peptides in SHK-1 cells

**Figure 8:**
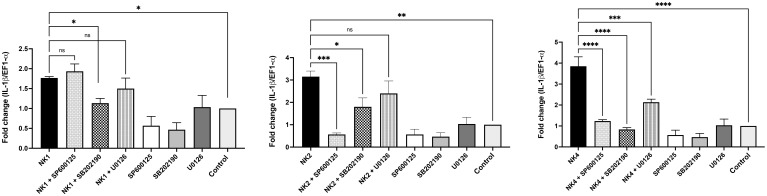
Activation of the MAPK signaling pathway by SsNK-lysin-derived peptides in *S. salar* HKLs. HKLs were pretreated for 2 hours with 10 μM of SB202190 inhibitor (p38 inhibitor), U0126 inhibitor (mitogen-activated protein kinase 1/2 (MEK1/MEK2) inhibitor) or SP600125 inhibitor (JNK inhibitor). As a negative control, cells were incubated with 0.1% DMSO as a vehicle for 2 hours. Cells were then incubated with 50 μM of NK1, NK2, NK4, or medium for 12 hours. The IL-1β relative expression was analyzed by qRT-PCR. The expression of the mRNA was analyzed as 2^−ΔΔCT^ relative quantification. The comparative threshold cycle values were normalized for EF-1α. Data were expressed as the means ± S.D. of three independent experiments, each in triplicate. Data were analyzed by ANOVA followed by Šidák’s multiple comparisons test (* p<0.05; ** p<0.01; ***p < 0.001; **** p<0.0001). Šídák method performs simultaneous joint pairwise comparisons for all possible pairwise combinations of means.

To investigate the relationship between the activation of the MAPK pathways and IL-1β induction, specific inhibitors of the MAPK pathways were used. The expression of IL-1β was significantly abrogated when HKLs were treated with NK4 in the presence of inhibitors, including SB202190, U0126, and SP600125 (p38, ERK1/2, and JNK inhibitors, respectively) (**Figure 8**). In contrast, leukocytes treated with NK1 only in the presence of SB202190 inhibitor significantly abolished the expression of IL-1β (**Figure 8**). On the other hand, the expression of IL-1β was significantly abrogated when HKLs were treated with NK2 in the presence SB202190 and SP600125 inhibitors (**Figure 8**). The results suggest that activation of the MAPK pathways is not only induced by NK-lysin-derived but is also necessary for the peptides-induced immune response.

### 3.8 SsNK-lysin-derived peptides regulate PAMPs-induced cytokine expression

**Figure 9:**
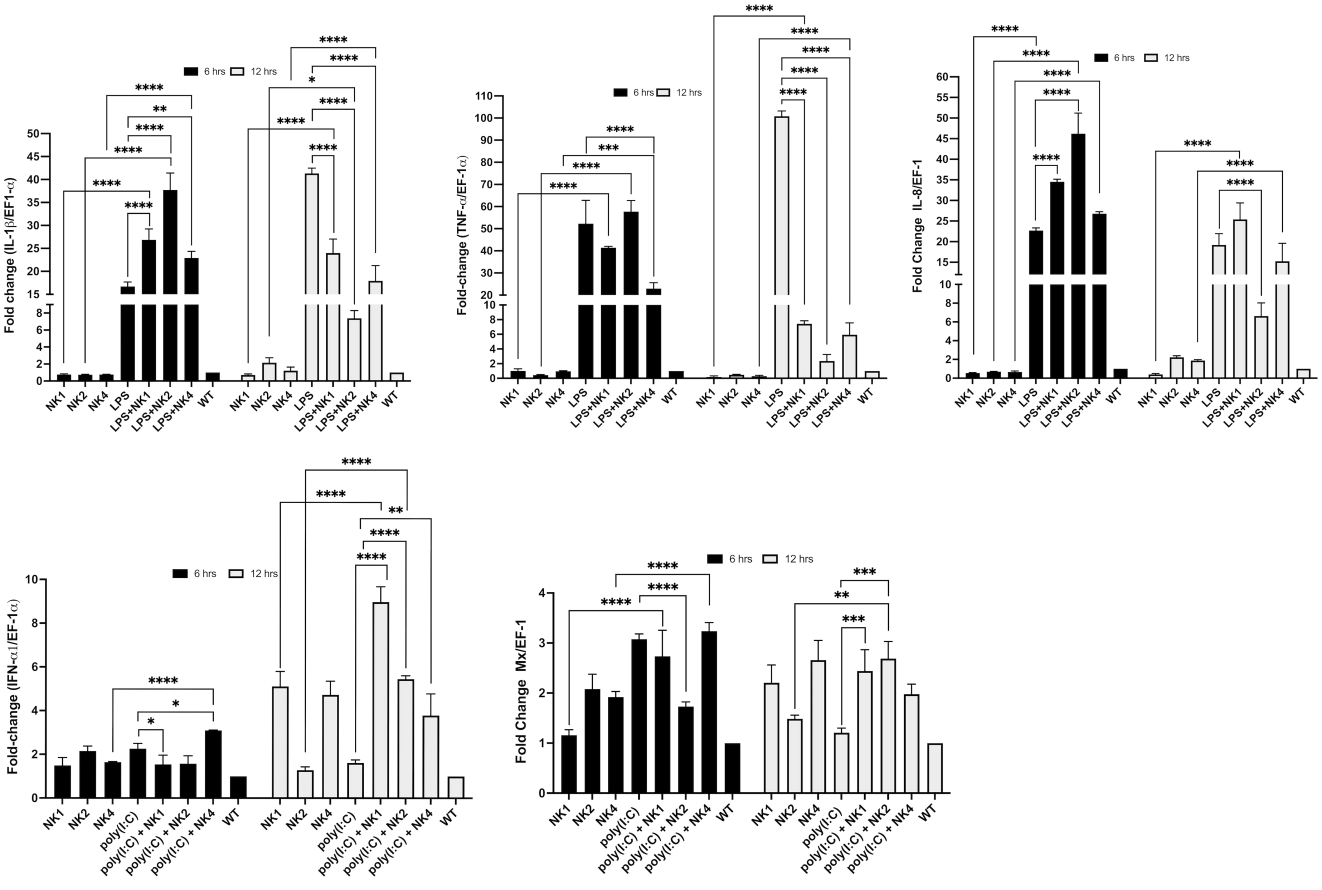
Modulation of LPS- or poly(I:C)-induced immune responses by co-administration of SsNK-lysin-derived peptides. SHK-1 cells were co-stimulated with 50 μM of SsNK-lysin-derived peptides (NK1, NK2, and NK4) and 1 µg/mL lipopolysaccharide or 1 µg/mL poly(I:C) for 6 and 12 hours. In addition, cells were treated with only LPS, poly(I:C), or peptides. Cells with culture medium alone were included as a negative control. The relative expressions of TNF-α, IL-1β, and IL-8 as LPS-induced responses and of IFN-1α and Mx involved in the antiviral response induced by poly(I:C) were determined by qRT-PCR. The expression of the mRNA was analyzed as 2^−ΔΔCT^ relative quantification. The comparative threshold cycle values were normalized for EF-1α. Data were expressed by the means ± S.D. of three independent experiments, each analyzed in triplicate, and by ANOVA followed by Šidák’s multiple comparisons test (* p<0.05; ** p<0.01; ***p < 0.001; **** p<0.0001). Šídák method performs simultaneous joint pairwise comparisons for all possible pairwise combinations of means.

We evaluated the immunomodulatory properties of the SsNK-lysin-derived peptides through their modulation of LPS and poly(I:C)-induced response in the SHK-1 cell line. SHK-1 cells were stimulated with LPS or poly(I:C) in the presence or absence of NK1, NK2, or NK4. The effect of SsNK-lysin-derived peptides on genes related to inflammatory and antiviral responses is shown in **Figure 9**. We found that the LPS challenge significantly elevated the expression levels of inflammatory cytokines such as the IL-1β, TNF-α, and IL-8 in the SHK-1 cells at 6 and 12 hrs post-treatment. However, co-treatment of LPS and NK-lysin-derived peptides significantly decreased the IL-1β and TNF-α expression levels at 12 hrs post-treatment. At 12 hrs, only the co-treatment of LPS and NK2 showed a significant decrease in the IL-8 expression level. NK1 and NK4 showed no significant change in the IL-8 expression level in the presence of LPS. At 6 hrs, the co-treatment of LPS and NK-lysin-derived peptides increased the IL-1β and IL-8 expression levels compared to the group treated with LPS alone. The co-treatment of NK1 or NK2 with LPS for 6 hrs didn’t show significant differences in the TNF-α expression level. However, the co-treatment of NK4 and LPS for 6 hrs significantly reduced the TNF-α expression level compared with LPS alone.

Besides, we determine if co-treatment with NK-lysin-derived peptides modulated the antiviral immune response to poly(I:C), a synthetic ligand of TLR3, and viral mimic in SHK-1 cells. The expression levels of the IFN-α1 and Mx genes were analyzed at 6- and 12 hours post-treatment. Poly(I:C) upregulated the expression of IFN-α1 and Mx only at 6 hrs post-treatment (**Figure 9**). Co-incubation with NK-lysin-derived peptides for 12 hrs significantly increased the transcription of IFN-α1 and Mx compared to poly(I:C) treated cells (**Figure 9**). At 6 hrs, the co-treatment of NK2 and poly(I:C) significantly reduced the Mx expression level. NK1 and NK4 didn’t affect the Mx expression level in the presence of poly(I:C). In addition, NK1 and NK2 reduced the IFN-α1 transcription, while NK4 significantly increased this level in the presence of poly(I:C).

### 3.9 Effects of SsNK-lysin-derived peptides on phagocytic activity in *Salmo salar* head kidney leucocytes

**Figure 10:**
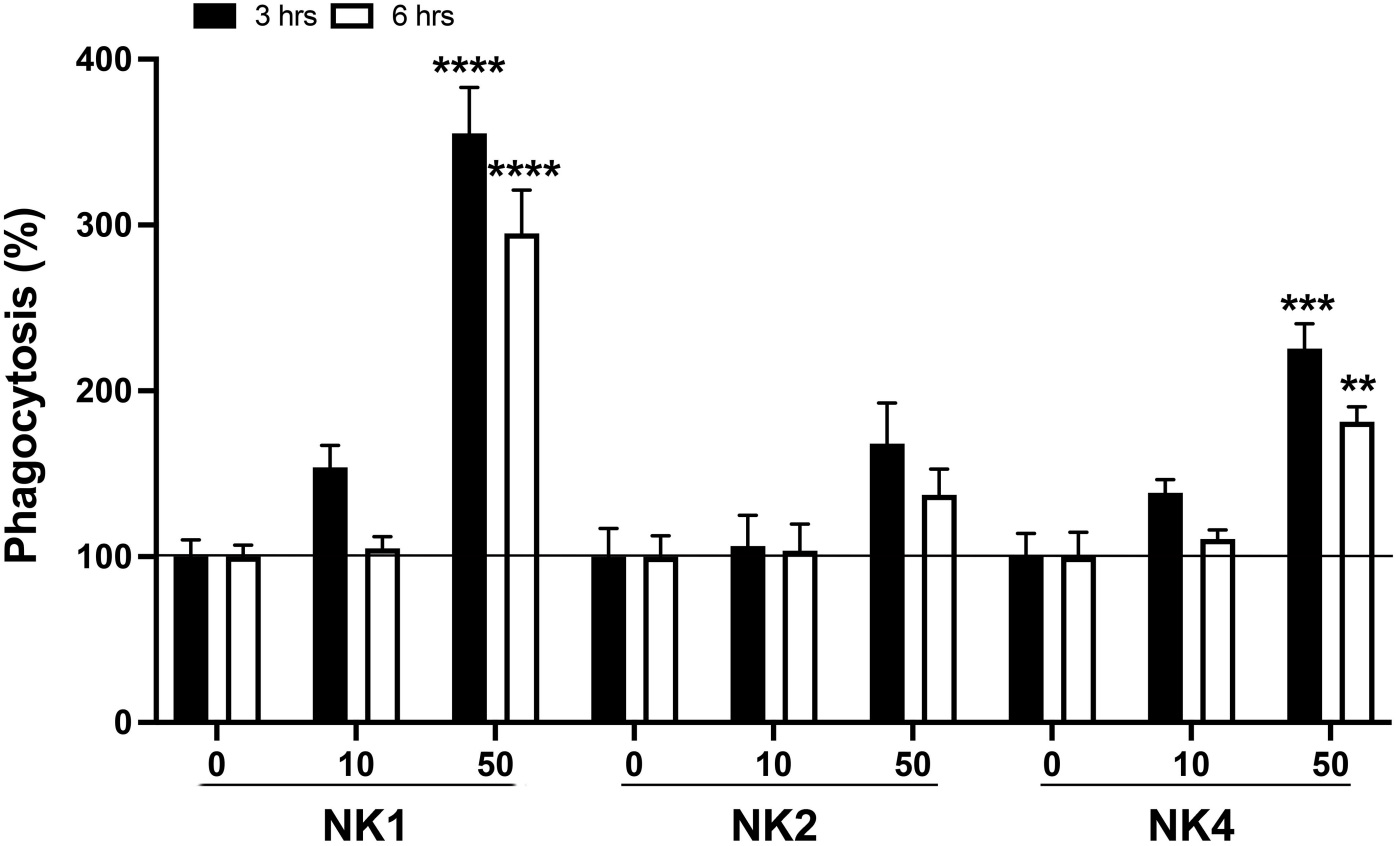
Effects on phagocytic activity of *S. salar* head kidney leukocytes (HKL). HKLs were incubated with pHrodo Green-conjugated *E. coli* bioparticles in the absence or presence of SsNK-lysin-derived peptides (NK1, NK2, and NK4) at 0, 10, and 50 μM. Phagocytosis in the absence of peptides was set at 100%. Data were expressed as percentages relative to cells incubated with bioparticles alone. Data represent means ± S.D. of three independent experiments performed in triplicate. Data were analyzed by ANOVA followed by Šidák’s multiple comparisons test. Asterisks indicate statistically significant differences compared to phagocytosis in the absence of stimuli (** p<0.01; ***p < 0.001; **** p<0.0001). Šídák method performs simultaneous joint pairwise comparisons for all possible pairwise combinations of means.

The phagocytic activity of the head kidney cells treated with Salmo salar NK-lysin-derived peptides was investigated (**Figure 10**). The phagocytosis rate of the head kidney cells treated with NK1 and NK4 at 50 μM was significantly upregulated at 3 and 6 hrs post-stimulation compared to that of the control (**Figure 10**). The NK1 showed the best results in the stimulation of phagocytic activity.

### 3.10 Cytokine expression profile in the head kidney of *Salmo salar* injected with SsNK-lysin derived peptides

**Figure 11:**
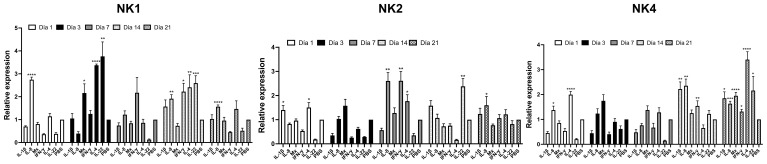
Relative transcriptional level of the immune-related gene in fish injected with *Salmo salar* NK-lysin-derived peptides at 1, 3-, 7-, 14- and 21 days post-injection. mRNA expression in Atlantic salmon’s head kidney after intraperitoneal injection of NK-lysin-derived peptides. The graph shows the cytokine fold induction compared to the control group, i.e., fish injected with phosphate-buffered saline. Data were expressed the means ± S.E. and analyzed by ANOVA followed by Dunnett’s multiple comparisons test (* p<0.05; ** p<0.01; ***p < 0.001; **** p<0.0001) (n = 5). The Dunnett *post hoc* test compared means from experimental groups against a control group mean.

In the *in vivo* experiment, the immunomodulatory effects of synthetic NK-lysin-derived peptides were evaluated. To this end, *Salmo salar* (50 g) were ip injected with NK-lysins-derived peptides (20 μg per fish). The control group was only injected with 100 μL PBS. At 1-, 3-, 7-, 14- and 21 days post-injection, fish (n = 5 per treatment group per time point) were euthanized, and head kidneys were collected for analysis of immune responses by RT-qPCR. The immune-related genes (IL-8, Mx, IL-4, IL-22, and IFN-γ) were upregulated in the head kidney of the NK1-injected group when compared with the control one (**Figure 11**). IL-8 expression was increased at day 1, 14 and 21 (2.7-, 1.9- and 1.6-fold change, respectively). Mx expression was increased at day 3 (2.2-fold change). IL-4 expression was increased at days 3 and 14 (3.4- and 2.4-fold change, respectively). IL-22 expression was increased at days 3 and 14 (3.8- and 2.6-fold change, respectively). IFN-γ expression was increased at day 14 (2.2-fold change) (**Figure 11**). When the fish were injected with NK2, the immune-related genes (IL-1β, IL-8, IL-4, IL-22, and IFN-γ) were upregulated in the head kidney (**Figure 11**). IL-1β expression was increased only at day 1 (1.4-fold change). IL-8 expression was increased at days 7 and 21 (2.6- and 1.6-fold change, respectively). IL-4 expression was increased at days 3 and 7 (1.5- and 1.8-fold change, respectively). IL-22 expression was increased at day 14 (2.4-fold change). IFN-γ expression was increased at day 7 (2.6-fold change) (**Figure 11**). The immune-related genes (IL-1β, IL-8, Mx, IL-4, IL-22, and IFN-γ) were upregulated in the head kidney of NK4-injected group when compared with the control one (**Figure 11**). IL-1β expression was increased at days 14 and 21 (2.2- and 1.9-fold change, respectively). IL-8 expression was increased on days 1, 14, and 21 (1.4-, 2.4- and 1.6-fold change, respectively). Mx expression was increased at day 21 (1.9-fold change). IL-4 expression was increased at days 1 and 21 (2- and 3.4-fold change, respectively). IL-22 expression was increased at day 21 (2.2-fold change). IFN-γ expression was increased at days 14 and 21 (1.5- and 1.3-fold change, respectively) (**Figure 11**).

## 4 Discussion

Previously, we identified four putative novel NK-lysin-like peptides from *S. salar* based on the EST database (17). We searched the GenBank gene databases for additional putative *NK-lysin* sequences in the present study. As a result, two additional *NK-lysin* coding transcripts were identified in Atlantic salmon. Besides, we identified the genes for the six NK-lysin-like peptides. The results showed that *NK-lysins 1, 2, 3, 5, and 6* are located in contiguous regions on chromosome 1, whereas the gene for *NK-lysin 4* is on chromosome 9. Similar results were observed in studies of NK-lysin sequences from *C. carpio* (20) and *O. mykiss* (22), where five genes were identified as clustered on the same chromosome, and a sixth gene was found on a different chromosome. The genes located on a different chromosome are also clustered on a more distant branch of the phylogenetic tree. At the same time, the other sequences clustered on the same branch of the tree are located on the same chromosome (22). This evidence supports the hypothesis that sequences on the same chromosome are duplicated genes derived from an ancestral gene, which is associated with gene diversification at the functional and tissue expression levels.

The two additional NK-lysin-coding transcripts reported here have a conserved signal peptide, the Saposin domain, and the six conserved cysteine residues. In addition, the six *S. salar* NK-lysins contain at least an additional cysteine residue conserved among them. SsNK-lysin-1 and SsNK-lysin-6 have eight cysteine residues in the mature peptide; one is absent in the rest of the *S. salar* NK-lysin sequences. On the other hand, SsNK-lysin-5 also has eight cysteine residues in the mature peptide, but one is only present in this sequence. The results obtained for *S. salar* are similar to what happened in *O. mykiss* (22). Recently, six genes for rainbow trout NK-lysin were identified. The rainbow trout nkl1-3 sequences have seven cysteine residues conserved with *S. salar*. In addition, the SsNK-lysin-4 and Omnkl1 sequences have, in the signal peptide, an additional cysteine residue conserved between them. The presence of the conserved cysteine residues and the Saposin-B domain suggests the existence of six NK-lysins in *S. salar*.

Although SsNK-lysins contain more than six cysteine residues, by Disulfide by Design 2 server, these molecules were predicted to form 3 conserved disulfide bonds. The parameter of main interest in this analysis was the energy expressed in kcal/mol since lower energy indicates a higher probability of disulfide bond formation. This relationship is because lower energy suggests greater stability in the conformation of disulfide bonds, which favors their formation in the protein structure. Besides, the hypothetical 3D structures revealed that the SsNK-lysin peptides possess the four/five-helical-bundle structure observed in the family of saposin-like proteins (19, 22).

The presence of more than one copy of NK-lysin in *S. salar* agrees with that obtained in other fish species, such as *O. mykiss* (22), *D. rerio* (19), *I. punctatus* (15), and *C. carpio* (20). However, other teleosts and higher vertebrates possess only one NK-lysin/granulysin (16). This diversity could indicate a specialization of different proteins in different functions.

The phylogenetic study suggests that the sequences identified from *S. salar* are more closely related to the nkl1, nkl2, and nkl3 sequences from *O. mykiss* and are clustered in the same clade, which is consistent with the results obtained by Ma. H et al., 2021 (22), where the *O. mykiss* nkl1, nkl2, and nkl3 sequences are more closely related. In agreement with their sequence homology, this evolutionary closeness between *S. salar* NK-lysin orthologous sequences could be related to whole genome duplication events (56–59), where the sequences identified here could have originated from a common ancestor. Therefore, multiple sequence alignment and phylogenetic analyses suggest that the proteins described here belong to the NK-lysin family.

NK-lysin are molecules of up to 100 amino acids, which makes their chemical synthesis difficult. For this reason, short NK-lysin-derived peptides with immunomodulatory and antimicrobial activities have been studied. In a previous study, we designed two 27 amino acid peptides derived from SsNK-lysin 1 and SsNK-lysin 2. This design was based on sequence alignments between NK-lysin identified in *S. salar* and NKLP27, a peptide derived from NK-lysin of *C. semilaevis* (17). In the present study, NK4, derived from SsNK-lysin 4, was designed based on *S. salar* NK1 and NK2. The sequences of the designed peptides correspond to the α-helices H2 and H3 of the NK-lysin SapB domain. This domain is essential in the antibacterial activity of NK-lysins (15, 17, 24, 33, 60–62).

It has been demonstrated that NK-lysins from teleost fish possess antimicrobial activities against various microorganisms (16, 18, 24, 28, 32, 36, 63, 64). However, no studies show these peptides’ antimicrobial effect against *P. salmonis* and *F. psychrophilum*. These two critical pathogens cause substantial economic losses in Chilean salmon aquaculture. Here, we evaluated the antibacterial activities of *Salmo salar* NK-lysin-derived peptides against *P. salmonis* and *F. psychrophilum*. NK1 and NK4 demonstrated antibacterial activity against the two Gram-negative bacteria. However, NK2 was only active for F. *psychrophilum*. These results suggested that these peptides could be efficacious in eliminating such bacteria.

The ability of AMPs to lyse eukaryotic cells is one of the significant obstacles to the use of AMPs. In the present work, we evaluated the hemolytic activity of synthetic peptides derived from NK-lysin. As a result, we obtained that NK1 was the most hemolytic peptide in human and fish erythrocytes, compared to NK2 and NK4. This peptide, compared to the other two, has the highest net charge (+6) and hydrophobic moment (0.53) and an intermediate value of hydrophobicity (0.07). Several studies show that the hemolytic activity of α-helical AMP is related to the hydrophobic characteristics of the peptide (65–67). Other studies have shown that net charge modulates the specificity and efficacy of these peptides’ antimicrobial and hemolytic activity (65, 68). In addition, the increase in hydrophobic moment, representing a quantitative measure of amphipathicity, increases hemolytic activity (65, 69). However, it is essential to note that there is no single factor that determines the antimicrobial and cytotoxic activity of an AMP; somewhat, these activities are influenced by a combination of factors such as sequence, net charge, hydrophobicity, and position of cationic residues (70). There are relatively few studies evaluating the hemolytic activity of NK-lysin-derived peptides. Most of these studies show low hemolytic activity for these peptides (15, 62, 71–75).

The most relevant immune tissues in teleost fish are the thymus, head kidney, caudal kidney, skin, gills, liver, spleen, and gut. The gills are mucosa-associated lymphoid tissues constituting an essential physical barrier and constantly being exposed to pathogens. Therefore, many immune-related molecules can be detected in the gills. In addition, the head kidney, and spleen are important sites of immune system activity and play a key role in defense against pathogen invasion (18). The NK-lysin protein has been extensively studied in mammals (76, 77). In addition, the most abundant constitutive expression of NK-lysin was identified in lymphoid tissues/cells in mammals (13, 76, 78, 79). In some teleost fish, the higher expression of NK-lysins has been detected in the gills, spleen, and/or head kidney (18, 20, 21, 80, 81). Under normal physiological status, the *S. salar NK-lysins 1, 2*, and *4* exhibit different expression patterns and abundances. However, the higher expression of all *SsNK-lysin* analyzed had been detected in gills, spleen, and/or head kidney. In rainbow trout, a salmonid species, *nkl1* and *nkl3* transcripts were expressed in all tissues examined. The *nkl1* showed the highest expression levels in the gills and spleen, whereas *nkl3* showed higher expression levels in the gills (22). The distribution of *nkl2* and *nkl4* transcripts was mainly restricted to the central nervous system (brain and pineal gland) and oocytes, but expression levels were generally low (22). In summary, *S. salar NK-lysins 1, 2*, and *4* are mainly expressed in immune tissues, suggesting they play a role in host immune defense.

In the present study, we showed that *S. salar* NK-lysin-derived peptides (NK1, NK2, and NK4) are potent stimulants of *Salmo salar* head kidney leukocytes and can up-regulate many cytokines from the innate and adaptive immune response. NK-lysin and their derived peptides modulating gene expression have been investigated previously in fish, but with only a limited number of cytokine genes studied (17, 30, 82), and the studies of immunomodulatory functions of NK-lysin in salmonids are scarce (17, 22). In our research, NK-lysin-derived peptide treatments upregulated most immune-related genes analyzed. NK1 and NK4 have the most prominent effects in the induction of pro-inflammatory cytokines (IL-1β, TNF-α and IL-8). Besides, these peptides increased the expression of T_H_1 cytokine IFN-γ and anti-inflammatory cytokine IL-10 and TGF-β. Although NK2 induces the expression of most of the genes analyzed, its effect is more modest compared to NK1 and NK4. It has been demonstrated that in addition to NK-lysins direct antimicrobial ability, these molecules may also have regulatory effects on immune cells. One study showed that a chicken NK-lysin-derived peptide (cNK-2) induced the expression of CCL4, CCL5, and IL-1β in HD11 and CCL4 and CCL5 in primary chicken monocytes (83). In addition, human granulysin increased the proinflammatory cytokines IL-6, IL-8, TNF-α, and IL-12 in T helper cell populations (84). In addition, we reported the effects of NK1 and NK2 on the expression of IL-1β, IL-8, and IFN-γ in head kidney leucocytes after 4 hours of treatment (17). There are some differences between our previous results and those obtained in the present work. These differences may be due to the different treatment times used and the fact that the assays were performed on primary cultures of the anterior kidney from different fishes. Therefore, the immune responses may vary according to genetic differences in the animals and differences in intrinsic factors such as sex, age, and immunological history (85).

Mitogen-activated protein kinases (MAPKs) are serine/threonine kinases conserved from yeast to mammals (86–88). In vertebrates, MAPKs contain three subfamilies of protein kinases: extracellular signal-regulated kinases (ERKs), c-Jun NH2-terminal kinases (JNKs), and the p38 family (89, 90). The MAPK signaling pathway is essential in innate and adaptive immunity and involves various cellular functions such as inflammation, cell differentiation, proliferation, and cell death (91). Besides, MAPK proteins induce the transcription of cytokines and chemokines (90–92). Since the MAPK pathway plays a central role in immunity and mediates the production of chemokines and cytokines, we evaluated whether NK-lysin-derived peptides induce IL-1β expression through this signaling pathway in HKLs from *S. salar*. According to our results, NK1 mediates the induction of IL-1β expression through the p38 MAPK pathway. NK4 induces IL-1β expression through p38, ERK1/2, and JNK MAPKs, whereas NK2 does so through p38 and JNK MAPKs. Our results are consistent with previous studies that reported the activation of MAPK signaling pathways by antimicrobial peptides or host defense peptides (HDP). LL-37 signals through induction of phosphorylation of MAPK proteins, ERK1/2, and p38, in peripheral blood-derived monocytes and a human bronchial epithelial cell line (HBE) (93). At least two of the immunomodulatory properties of LL-37 increased IL-8 secretion and transcription of the chemokine’s monocyte chemoattractant protein 1 (MCP-1), MCP-3, and IL-8, are dependent on activation of p38 and ERK1/2 kinases (93). Besides, Kim et al. (2017) demonstrated that chicken NK lysin-derived cNK-2 stimulates the MAPK pathway and induces the expression of proinflammatory cytokines and chemokines (83). On the other hand, it was demonstrated that chicken defensin (AvBD8) mediates the immune response through the MAPK signaling pathway by phosphorylating ERK1/2 and p38 signaling molecules (94). Our work is the first study in fish to demonstrate that NK-lysin-derived peptides modulate the immune response through activation of the MAPK signaling pathway in the *S. salar* HKLs.

In the present work, we determined the effect of *S. salar* NK-lysin-derived peptides on the response of SHK-1 cells to the pathogen-associated molecular patterns (PAMPs), LPS, and poly(I:C). We demonstrate that the peptides modulate the immune response to LPS by inhibiting the expression of pro-inflammatory cytokines such as IL-1β and TNF-α at 12 hours of stimulation. To our knowledge, no studies in fish describe the regulation exerted by NK-lysin on the immune response induced by PAMPs. The only report that exists for NK-lysin is in chicken. Kim et al. (2017) demonstrated that a chicken NK-lysin-derived peptide (cNK-2) modulates the LPS-induced inflammatory response with reduced expression of the proinflammatory cytokine IL-1β in HD11 cells and monocytes (83). Numerous studies demonstrate that antimicrobial peptides inhibit the proinflammatory responses produced by various Toll-like receptor ligands, including LPS, by reducing proinflammatory mediators (95–101).

On the other hand, we demonstrate that the peptides modulate the immune response to poly(I:C) by enhancing the expression of antiviral genes such as IFN-α1 and Mx at 12 hours of stimulation. Studies describing the role of antimicrobial peptides in regulating the poly(I:C)-induced immune response are more controversial. For example, LL37 enhanced poly(I:C)-induced IL-6 and IFN-β levels when compared to poly(I:C) alone in bronchial epithelial cells (102). Besides, the treatment of human PBMCs with either LL37 or poly(I:C) had modest effects on IL-1α, MCP-1, and IP-10 levels, but the addition of LL37 and poly(I:C) increased the levels of IL-1α, MCP1, and IP10 by at least ten-fold above the levels seen with either poly(I:C) or LL37 alone (102). These results demonstrate that LL37 and poly(I:C) combination enhances cytokine production in primary cells as in immortalized cell lines. Another study evaluated the effect of poly(I:C) in the RAW 264.7 cell line in the presence or absence of the peptide BMAP-28 (Bovine Myeloid Antimicrobial Peptide of 28 predicted amino acid residues) (97). The poly(I:C) increased IFN-β gene expression in cells stimulated for 3 h. BMAP-28 significantly increased this response. Although poly(I:C) alone was a weak inducer of IL-1β, TNF-α, and IL-6 genes compared with the effect produced by LPS, its effect was detectably increased in the presence of BMAP-28 (97). Another study determined the effect of the human defensin hBD3 on the response of primary macrophages to poly(I:C). As a result, they obtained that poly(I:C) in the presence of hBD3 has an exacerbated IFN-β response and decreases CXCL10 production, *in vitro* and *in vivo*, both in mice and human primary cells (103). The results of other studies contradict those described above (101, 104). The effect of the antimicrobial peptide LL-37 on the proinflammatory responses of human gingival fibroblasts (HGF) stimulated with microbial Toll-like receptor (TLR)-stimulating compounds such as poly(I:C) was evaluated. LL-37 suppressed poly(I:C)-induced IL-6, IL-8 and CXCL10 expression (101). Another study showed that RAW 264.7 macrophages produced TNF-α, IL-6, and IL-1β after stimulation with poly(I:C), and this stimulation was inhibited in a dose-dependent manner by mCRAMP or LL37 peptides. This study showed that TLR3 signaling was not enhanced but drastically inhibited by LL37 or mouse cathelicidin-related antimicrobial peptide (mCRAMP) in macrophages, microglial cells, and dendritic cells and that the inhibition correlated with the formation of a strong complex between antimicrobial peptides and poly(I:C), which partially inhibited the binding of poly(I:C) to TLR3 (104). Considering the above results, additional studies are required to investigate further the effect of NK-lysin peptides on the PAMPs-induced response and their mechanism of action. It is important to note that the antiviral genes IFN1a and Mx were chosen as there are studies, both *in vitro* and *in vivo*, demonstrating that poly(I:C) induces the expression of these genes in several species (103, 105–113). In most previous *in vitro* studies, they used a poly(I:C) dose of 10, 25, or 50 μg/ml (101, 103, 111–113). The effect of poly(I:C) when using these doses is far superior to that obtained in the present work. However, to better evaluate the immunoregulatory effects of peptides on poly(I:C)-induced antiviral gene expression, we decided to use a much smaller dose (1 μg/ml) in our assays. We suggest that the dose is the fundamental reason why the effect of poly(I:C) is much lower than reported in previous studies.

Phagocytosis in vertebrates has been recognized as a critical component of innate and adaptive immune responses to pathogens and is crucial for tissue homeostasis and remodeling (114). The cells responsible for phagocytosis in teleost fish include neutrophils, monocytes of the hemopoietic organs, and free and fixed macrophages of the spleen and kidney. These cells achieve pathogen clearance through respiratory burst and reactive oxygen species production (115). In teleost fish, the cephalic kidney is an essential lymphoid organ in the proliferation and differentiation of B lymphocytes (116) and macrophage production (117). It acts as a site for capturing and processing pathogens and foreign substances (118). In the present work, we evaluated the effects of NK-lysin-derived peptides over phagocytic activity in head kidney leukocytes from *S. salar*. We obtained that the phagocytosis rate of the head kidney cells treated with NK1 and NK4 at 50 μM was significantly upregulated at 3 and 6 hrs post-stimulation compared to that of the control. The previous results concerning the phagocytic activity of fish NK-lysin peptides are controversial. A 2019 study investigated the effects of an NK-lysin-derived peptide from *Boleophthalmus pectinirostris* (BpNKLP40) on phagocytosis in monocytes/macrophage (MO/MФ) of *B. pectinirostris* and observed that treatment with BpNKLP40 had no significant effect on phagocytosis of FITC-*E. tarda* by these cells (24). Another study in Nile tilapia (*O. niloticus*) evaluated whether the phagocytic activities of MO/MФ from anterior kidney leukocytes could be enhanced by stimulation with tilapia NK-lysin protein. For this, cells were incubated with fluorescent microspheres (0.5 and 1.0 μm microspheres) and bioparticles (*S. agalactiae* and *A. hydrophila*) for analysis by flow cytometry. The result suggests that NK-lysin protein enhances the phagocytic activities of MO/MΦ. Moreover, this enhancement in phagocytosis induced by NK-lysin protein was dose-dependent (82). In addition, the phagocytic activity of leukocytes derived from the anterior kidney and spleen of *P. olivaceus* exposed to rPoNKL was evaluated. As a result, it was observed that this activity did not vary significantly compared to untreated cells (119).

The antimicrobial peptides can be used as immunostimulants or molecular adjuvants. However, to use an antimicrobial peptide as an immunostimulant or a vaccine adjuvant in a fish species, it is necessary first to understand the events of the host response, the activation of inflammatory responses, and adaptative defense mechanisms. Among the potential fish AMPs, NK-lysin-derived peptides have shown direct or indirect antibacterial, antiviral, and antiparasitic activities (15, 16, 19, 33, 40, 120–123). However, only a few studies have evaluated the immunomodulatory activity of fish NK-lysin *in vivo* (16, 27, 33, 124). We are unaware of any other reports directly showing the effects of NK-lysin over cytokines from the adaptive immune response. In the present study, we used a targeted approach to determine the impact of *Salmo salar* NK-lysin-derived peptides on selected immune-relevant genes to gain in-depth information on these events in our model specie, the Atlantic Salmon (*Salmo salar*). At varying degrees of intensity and at different post-injection times, *S. Salar* NK-lysin-derived peptides induce the expression of pro-inflammatory cytokine IL-1β, IL-8; antiviral protein Mx; T_H_1 cytokine IFN-γ (125); T_H_17 cytokine IL-22 (125) and T_H_2 cytokine IL-4/13 (125).

Our results in Atlantic salmon show that innate and adaptive immune responses are activated following NK-lysin-derived peptides injection, activating multiple pathways. All these data suggest that NK-lysin can be used as a potent immunostimulant or a vaccine adjuvant in fish aquaculture.

## Data availability statement

The original contributions presented in the study are included in the article/**Supplementary Material**. Further inquiries can be directed to the corresponding author.

## Ethics statement

The animal study was approved by Universidad de Concepcion CEBB 693-2020. The study was conducted in accordance with the local legislation and institutional requirements.

## Author contributions

LO: Conceptualization, Data curation, Formal analysis, Investigation, Methodology, Writing – review & editing. CC: Conceptualization, Data curation, Formal analysis, Investigation, Methodology, Writing – review & editing. CM-F: Conceptualization, Methodology, Validation, Formal analysis, Investigation, Visualization, Writing – original draft, Writing – review & editing. SS: Investigation, Methodology, Writing – review & editing. MS: Formal analysis, Investigation, Supervision, Writing – original draft, Writing – review & editing. MV: Methodology, Validation, Formal analysis, Investigation, Writing – review & editing. AV: Conceptualization, Investigation, Supervision, Writing – review & editing. NA: Methodology, Validation, Formal Analysis, Investigation, Writing – review & editing. RM: Conceptualization, Investigation, Supervision, Writing – review & editing. AA: Investigation, Methodology, Supervision, Writing – review & editing. NP: Investigation, Methodology, Supervision, Writing – review & editing. EP: Investigation, Methodology, Writing – review & editing. NS: Investigation, Methodology, Writing – review & editing. AR: Conceptualization, Methodology, Resources, Data curation, Writing – review & editing, Funding acquisition. PR: Investigation, Methodology, Writing – review & editing. EL: Investigation, Methodology, Writing – review & editing. FR: Investigation, Methodology, Writing – review & editing. OS: Conceptualization, Methodology, Resources, Data curation, Writing – review & editing. JT: Conceptualization, Methodology, Resources, Data curation, Writing – review & editing. JA: Conceptualization, Methodology, Resources, Writing – original draft, Writing – review & editing, Supervision, Funding acquisition.
