# Supplementary material for: New insight into the biological activity of Salmo salar NK-lysin antimicrobial peptides
Source: Front Immunol. 2024 Apr 9;15:1191966. doi: 10.3389/fimmu.2024.1191966 (PMC11035819; doi:10.3389/fimmu.2024.1191966)
Supplement: Supplementary file 1 [file Table_1.docx]

**Supplementary material**

**Table 1.** Physicochemical properties of the three NK-lysin-derived peptides calculated using the Database of Antimicrobial Activity and Structure of Peptides (https://dbaasp.org/home).

| **ID** | **Hydrophobic Moment** | **Hydrophobicity** | **Net Charge** | **Isoelectric Point** | **Amphiphilicity Index** |
| --- | --- | --- | --- | --- | --- |
| NK1 | 0.53 | 0.07 | 6.00 | 10.56 | 0.94 |
| NK2 | 0.52 | -0.09 | 3.00 | 9.52 | 1.00 |
| NK4 | 0.51 | 0.09 | 5.00 | 10.32 | 0.93 |
